# Divergent Genomic Adaptations in the Microbiomes of Arctic Subzero Sea-Ice and Cryopeg Brines

**DOI:** 10.3389/fmicb.2021.701186

**Published:** 2021-07-22

**Authors:** Josephine Z. Rapp, Matthew B. Sullivan, Jody W. Deming

**Affiliations:** ^1^School of Oceanography, University of Washington, Seattle, WA, United States; ^2^Byrd Polar and Climate Research Center, Ohio State University, Columbus, OH, United States; ^3^Department of Microbiology, Ohio State University, Columbus, OH, United States; ^4^Department of Civil, Environmental and Geodetic Engineering, Ohio State University, Columbus, OH, United States; ^5^Center of Microbiome Science, Ohio State University, Columbus, OH, United States

**Keywords:** cryopeg, sea ice, metagenomics, metatranscriptomics, microbial ecology, hypersalinity, subzero temperature, cryosphere

## Abstract

Subzero hypersaline brines are liquid microbial habitats within otherwise frozen environments, where concentrated dissolved salts prevent freezing. Such extreme conditions presumably require unique microbial adaptations, and possibly altered ecologies, but specific strategies remain largely unknown. Here we examined prokaryotic taxonomic and functional diversity in two seawater-derived subzero hypersaline brines: first-year sea ice, subject to seasonally fluctuating conditions; and ancient cryopeg, under relatively stable conditions geophysically isolated in permafrost. Overall, both taxonomic composition and functional potential were starkly different. Taxonomically, sea-ice brine communities (∼10^5^ cells mL^–1^) had greater richness, more diversity and were dominated by bacterial genera, including *Polaribacter*, *Paraglaciecola*, *Colwellia*, and *Glaciecola*, whereas the more densely inhabited cryopeg brines (∼10^8^ cells mL^–1^) lacked these genera and instead were dominated by *Marinobacter*. Functionally, however, sea ice encoded fewer accessory traits and lower average genomic copy numbers for shared traits, though DNA replication and repair were elevated; in contrast, microbes in cryopeg brines had greater genetic versatility with elevated abundances of accessory traits involved in sensing, responding to environmental cues, transport, mobile elements (transposases and plasmids), toxin-antitoxin systems, and type VI secretion systems. Together these genomic features suggest adaptations and capabilities of sea-ice communities manifesting at the community level through seasonal ecological succession, whereas the denser cryopeg communities appear adapted to intense bacterial competition, leaving fewer genera to dominate with brine-specific adaptations and social interactions that sacrifice some members for the benefit of others. Such cryopeg genomic traits provide insight into how long-term environmental stability may enable life to survive extreme conditions.

## Introduction

The cryosphere, that portion of the planet where most of the water is frozen, includes more than half of land surfaces and 7% of the surface ocean globally (Vaughan et al., 2013). These frozen regions are incurring significant losses due to climate change (Fountain et al., 2012). Cryosphere components like permafrost, relatively stable features that have remained frozen for thousands or even millions of years (Froese et al., 2008), are warming now (Biskaborn et al., 2019; Nitzbon et al., 2020). Others, like sea ice, are inherently short-lived, with formation and melting occurring annually (Vaughan et al., 2013), yet recent losses in areal extent and volume, particularly for Arctic sea ice, are pronounced (Overland and Wang, 2013; Stroeve and Notz, 2018). Though largely frozen, these at-risk environments contain subzero hypersaline brines that provide interior liquid habitat for diverse microbial life of both ecological relevance and inherent curiosity for their unique adaptations to extreme conditions (Boetius et al., 2015). With continued warming, opportunities to study these microbes and their current roles *in situ* dwindle, yet the molecular microbial ecology of the cryosphere remains underexplored compared to other areas of environmental microbiology (reviewed by Deming and Collins, 2017). Direct comparisons between subzero brines that differ in age or stability are rare (Cooper et al., 2019), and have not yet included an exploration of genomic adaptations to long-term vs. fluctuating extreme conditions. The challenges are many, including safe accessibility, sampling without altering (melting) the habitat, and accounting for the complexities and heterogeneity of the micro-scale living spaces within ice, especially in sea ice (Junge et al., 2001), which is subject to seasonal extremes in temperature and salinity (Ewert and Deming, 2013, 2014) and thus fluctuating porosity (Golden et al., 2007; Petrich and Eicken, 2017).

The habitable space within sea ice or any frozen-water matrix exists in the form of hypersaline liquid inclusions, kept liquid well below 0°C due to freezing point depression by the concentration of dissolved salts (Petrich and Eicken, 2017) and, to a lesser extent, dissolved organics (Deming and Young, 2017). Despite subzero temperatures and high salinities, diverse microbial communities have been reported for even the most extreme cryosphere habitats, including wintertime sea ice at −28°C and 240 ppt salt (Collins et al., 2008, 2010). For a microorganism to inhabit a subzero brine its core cellular processes must be adapted to function under the extreme conditions of temperature and salinity. Classically, research has focused on cellular strategies to counteract these abiotic stressors, particularly through osmolytes (Kempf and Bremer, 1998; Ewert and Deming, 2014; Firth et al., 2016), chemical modification of the cell envelope (Chintalapati et al., 2004), pigment production (Dieser et al., 2010), and synthesis of cold-shock proteins and nucleic acid chaperones (Baraúna et al., 2017). Many organisms in frozen environments also synthesize extracellular polysaccharides (EPS or exopolymers), with chemical characteristics that provide cryoprotection through freezing-point depression or inhibition of ice recrystallization (Carillo et al., 2015) and osmoprotection (Krembs and Deming, 2008; Deming and Young, 2017).

Sea ice may be among the best studied of cryosphere habitats, with winter microbial communities reflecting the parent seawater prior to freezing (Collins et al., 2010) and characteristic Gammaproteobacteria and Bacteroidia reported consistently during spring and later seasons (Brown and Bowman, 2001; Eronen-Rasimus et al., 2016; Rapp et al., 2018). Such studies have been based mostly on 16S rRNA genes, whether from cultured bacteria or melted sea-ice samples; few have used metagenomic sequencing to explore the resident bacteria and archaea (Bowman et al., 2014; Yergeau et al., 2017). We are not aware of metagenomic analyses of microbial communities in subzero sea-ice brines obtained directly, without the compromise of first melting the ice.

In stark contrast to the transient brines of sea ice are the stable, ancient (late Pleistocene) and rarely studied brines of cryopegs, marine-derived sediment layers within permafrost (Gilichinsky et al., 2003, 2005) that contain discrete lenses of brine geophysically isolated at fairly constant temperature year-round, ranging between –9 and –11°C in Siberian cryopegs (Gilichinsky et al., 2005) and –6 and –8°C in Alaskan cryopegs (Colangelo-Lillis et al., 2016; Cooper et al., 2019). A recent first exploration of microbial community composition in cryopeg brines based on 16S rRNA genes, obtained from the same cryopeg system also studied here, revealed a less diverse and distinctive community structure than observed in sea-ice brines (Cooper et al., 2019), with concentrations of total and dividing cells greatly exceeding those in the sampled sea-ice brines (approximately 10^8^ vs. 10^5^ cells mL^–1^ and up to 29 vs. ∼ 5%, respectively; Table 1). Most of the sampled cryopeg brines were dominated by only a few taxa, in particular members of the genus *Marinobacter*, yet the specific adaptations enabling their success under the stably extreme conditions remain unknown.

**TABLE 1:** Sample characteristics including temperature, salinity, pH, organic matter and nutrient concentrations, prokaryotic cell numbers, and percent dividing cells (data compiled from Cooper et al., 2019; Zhong et al., 2020).

Parameter (unit)	Cryopeg brines					Sea-ice brines^*a*^		Sea-ice sections^b^	
Sample label	CBIW^c^	CBIW	CBIA	CB1	CB4^d^	SB^c^	SB^d^	SI3U	SI3L
Sampling year	2017	2018	2018	2018	2018	2017	2018	2017	2017
T (°C)	–6	–6	–6	–6	–6	–4	–3	–5	–3
S (ppt)	140	121	112	122	121	78	75	18	18
pH	6.6	–	–	6.6	6.6	–	7.2	–	–
POC (μg C mL^–1^)	23.8	29.0	–	145.1	46.3	0.24	0.22	0.20	2.53
PON (μg N mL^–1^)	3.92	5.32	–	23.99	6.98	0.03	0.04	0.03	0.34
C:N value	6.06	5.46	–	6.08	6.62	8.94	5.66	7.64	7.46
DOC (μM C)	3.00 × 10^4^	8.22 × 10^4^	–	1.02 × 10^5^	8.50 × 10^4^	4.46 × 10^2^	2.00 × 10^2^	2.27 × 10^2^	4.97 × 10^2^
pEPS (μM C)	1.44 × 10^4^	1.14 × 10^2^	–	1.63 × 10^3^	8.91 × 10^1^	1.90 × 10^0^	9.67 × 10^–1^	2.98 × 10^0^	2.93 × 10^1^
dEPS (μM C)	8.55 × 10^3^	1.25 × 10^4^	–	1.94 × 10^4^	1.98 × 10^4^	2.61 × 10^2^	bd	–	–
PO_4_ (μM)	1.94	0.68	–	1.22	0.6	1.8	1.6	0.21	0.19
NO_3_ (μM)	5.56	0.82	–	bd	13.6	0.15	3.63	0.31	0.87
NO_2_ (μM)	2.03	0.89	–	16.2	2.96	0.02	0.11	0.03	bd
NH_4_ (μM)	1.75 × 10^3^	1.17 × 10^3^	–	3.35 × 10^3^	4.52 × 10^3^	5.50 × 10^–1^	3.42 × 10^0^	4.60 × 10^–1^	bd
Prokaryotic cells (mL^–1^)	1.39 × 10^8^	1.22 × 10^8^	7.31 × 10^7^	9.57 × 10^7^	1.14 × 10^7^	2.22 × 10^5^	1.11 × 10^5^	7.68 × 10^4^	1.07 × 10^6^
Dividing cells (%)	–	1.3	2.5	1.9	29	–	4.68	–	–

*^a^Collected directly from “sackholes” in the ice. ^b^All values determined on melted ice samples and scaled to ice volume; scaling to brine volume (brine fraction of the ice) would increase values by an estimated factor of 2.5 for SI3U and 1.6 for SI3L (Cox and Weeks, 1983). ^c^Two size fractions of this sample were included for metagenomic analysis (see Supplementary Table 1 for details). ^d^Both metagenomic and metatranscriptomic analyses were performed. –, not determined; bd, below detection limit.*

Because the freezing process that concentrates salts within the brine inclusions also concentrates microorganisms and other particulate and dissolved constituents present in the source water, microbial cell numbers, as well as organic matter and nutrient concentrations in the brines, are elevated compared to their source. As a result, cell-to-cell contact rates within sea ice are much higher than in seawater (Collins and Deming, 2011), and would be even higher in a cryopeg brine containing 10^8^ cells mL^–1^ (Table 1). Higher cell concentrations and contact rates in a spatially constrained setting present additional challenges in the form of resource and space competition, yet they may also lead to genetic exchange and diversification, as several examples of genes relevant to cold or salt adaptation appear to be the result of horizontal gene transfer (Bowman, 2008; Collins and Deming, 2013; Feng et al., 2014; Zhong et al., 2020). To maintain or increase cell concentrations over time, microbes in subzero brines likely require versatile nutrient acquisition capabilities, given slow rates of diffusion in such viscous fluids (Showalter and Deming, 2021). While microbes in sea ice may be presented with sufficient organic matter from sea-ice algae during the spring and summer months, they must also survive the aphotic winter season. In cryopeg brines, where phototrophic primary production is absent, heterotrophic microbes may survive *via* the products of their extracellular enzymes (Showalter and Deming, 2021), hydrolyzing remnant plant and detrital material from the surrounding permafrost and ice-wedge environments (Iwahana et al., 2021), and *via* dead microbial matter, as proposed for oceanic subsurface marine sediments (Bradley et al., 2018), yet any metagenomic insights into survival mechanisms employed *in situ* are currently lacking.

Here we present a comparative metagenomic analysis of the taxonomic and functional diversity of microbial communities present in brines from first-year sea ice and in ancient cryopeg brines found deep within approximately 40,000-year-old permafrost (Meyer et al., 2010; Iwahana et al., 2021) near Utqiaġvik, Alaska. Both of these seawater-sourced habitats share the traits of subzero temperature and hypersalinity (–4 to –3°C and 75–78 ppt for the sea-ice brines at time of sampling, and –6°C and 112–140 ppt for the cryopeg brines; Table 1), yet they differ greatly in their environmental age and stability. We used metagenomic and selected metatranscriptomic sequencing to investigate how communities in these two extreme habitats are adapted to life under subzero and hypersaline conditions, and whether they are following similar or different strategies to overcome potential resource and space competition. Our overall aim is to improve the understanding of the impacts of environmental stability vs. transience on the adaptability of life to such extreme conditions.

## Materials and Methods

### Field Sampling

We collected samples near Utqiaġvik, Alaska, during two field seasons in May 2017 and May 2018. Landfast, first-year sea ice was sampled near the Barrow Sea Ice Mass Balance site, operated by the University of Alaska Fairbanks (Druckenmiller et al., 2009), at 71.2223°N, 156.3018°W in 2017 and 71.2238°N, 156.3083°W in 2018. At both times, the sites were covered with a layer of snow, 16–19 cm in 2017 and 6–10 cm in 2018, which we cleared prior to sampling activities. Air temperature at 1 m above the ice varied between –6 and –4°C in both years. Partial ice-core holes (sackholes) were drilled at 1-m distance from each other to a depth of 75 cm in 2017, when ice thickness was 117 cm, and to 55 cm in 2018, when ice thickness was 110 cm. Following Eicken et al. (2009), we covered the sackholes and allowed brine to drain into them for 3–5 h before collection. The first sampling of the sea-ice brine (SB), after sufficient volumes for all other sampling needs had accumulated, was for RNA extraction. We used a sterile syringe to withdraw 50 ml of brine from each of 10 individual sackholes and pooled the brines onto one Sterivex filter (MilliporeSigma), which we treated immediately on site with RNAlater^TM^ (Invitrogen, Thermo Fisher Scientific). For DNA analyses and all ancillary measurements, we then collected 20 L of SB by hand-pumping into an acid-washed cubitainer, first rinsed with sample brine.

In each year, we collected full-length sea-ice cores using a MARK II ice auger (Kovacs Enterprise) with a diameter of 9 cm. For two cores, vertical temperature measurements were made in the field at 5-cm intervals according to Eicken et al. (2009), from which we inferred the sackhole brine temperature of –4 and –3°C in both years (Table 1). Sackhole brine salinities were measured by hand-held refractometer, yielding salinities of 75–78 ppt in both years. In 2017, we sectioned one sea-ice core in the field to obtain the upper 25 cm (SI3U), middle 50–75 cm (SI3M) and lowermost 25 cm (SI3L) using a 70% ethanol-rinsed, custom-alloy bow saw. Each ice section was collected in a sterile Whirl-Pak bag. As SI3M failed subsequent sequencing efforts, it does not appear in this study.

Cryopeg brine (CB) was obtained through the Barrow Permafrost Tunnel, located at 71.2944 °N, 156.7153 °W and situated 6 m below the surface. The temperature inside the tunnel held at –6°C during our sampling work (Table 1). Brines were extracted from approximately 2 m below the tunnel floor through discrete boreholes, some previously established (Colangelo-Lillis et al., 2016) and others drilled for this study using a cleaned and ethanol-rinsed ice auger or SIPRE corer (as in Colangelo-Lillis et al., 2016). We used a hand pump and 1.5-m length of sterile Masterflex and Teflon tubing (Cole-Parmer, Vernon Hills, IL, United States) to collect samples in acid-washed 2-L vacuum flasks (initially autoclaved, then acid-washed and ethanol-rinsed between samples). Detailed borehole histories and sampling are provided in Cooper et al. (2019) and Iwahana et al. (2021). Sample names here are based on borehole name and filtration pore size (see below). During the two field campaigns we were able to obtain a total of six cryopeg brine samples for metagenomic analyses: one each from boreholes CB4, CB1 and CBIA and three from borehole CBIW, representing different years and filtration steps. Brine volumes were very limited, but we were able to collect sub-samples for RNA extraction from two of the boreholes (CB4 and CBIW), concentrating 100 ml on a Sterivex^TM^ filter (MilliporeSigma) and immediately stabilizing it with RNAlater^TM^ (Invitrogen, Thermo Fisher Scientific) while still in the tunnel. The metatranscriptome sequencing library for CBIW later failed sequencing efforts and no longer appears in this study.

We stored all samples in insulated coolers on site and during transport to cold rooms set to near *in situ* brine temperatures at the Barrow Arctic Research Center. Cryopeg brines were held at –6°C and sea-ice brines at –1°C until further processing within 2–8 h. Sea-ice sections were melted into a filter-sterilized (0.22-μm pore size) artificial sea salt solution (salinity of 32 g/L, Cat No. S9883, Sigma) at a 1:1 vol:vol ratio. For subsequent DNA extraction, maximum available volumes were filtered onto 47-mm GTTP Isopore filters (Supplementary Table 1), after accounting for sufficient minimum volumes to remain for basic sample characterization (Table 1). The sea-ice samples and one cryopeg brine sample (CBIW in 2017) were filtered sequentially through filters of 3.0 μm (CBIW_3.0) and 0.2 μm (CBIW_0.2) pore size to obtain operational microeukaryotic and prokaryotic fractions, respectively. Biomass from the other cryopeg brines was collected on a 0.2 μm pore size filter without prefiltration. The artificial sea salt solution was processed in parallel as a blank (Supplementary Figure 1). For subsequent RNA extraction, we incubated the Sterivex filters in RNAlater overnight at 4°C, subsequently transferring them to –20°C until extraction according to the manufacturer’s instructions.

### DNA and RNA Extraction

For DNA extractions, we used the DNeasy PowerSoil kit (QIAGEN, Germantown, MD, United States), following the manufacturer’s instructions. To ensure free movement of the samples during bead beating, we cut filters into stripes using sterilized scalpels and forceps, and performed two extractions per sample, each containing half a filter. Isolated DNA was eluted in 60 μl nuclease-free water (Thermo Fisher Scientific, Waltham, MA), and both extracts from the same filter were pooled and stored at –20°C.

Prior to any RNA work, we cleaned all working areas and equipment with 70% Ethanol, followed by a wipe with RNaseZap^TM^ (Thermo Fisher Scientific, Waltham, MA). For RNA extractions, we used the DNeasy PowerWater Sterivex Kit (Qiagen, Germantown, MD), following the manufacturer’s instructions, with the following modifications developed by the QIAGEN microbiome team that allowed recovering RNA from the filter membranes without the need to cut open the plastic casing holding the membrane: After removing RNAlater^TM^ from the casing using a sterile 3-ml syringe, we followed protocol, but added 20 μl of β-mercaptoethanol (βME) for every 880 μl of Solution ST1B in step 2. Further, we modified step 7 and incubated the filter unit at 70°C for 10 min, and in step 19 added 1.5 ml of Solution MR, as well as 1.5 ml of 100% ethanol. We eluted the isolated RNA in 50 μl of sterile RNase-free water (Thermo Fisher Scientific, Waltham, MA) and performed a subsequent DNA digest by adding 7 μl DNase buffer (10×) (Sigma-Adrich, St. Louis, MO), 10 μl DNase I recombinant (Sigma-Adrich, St. Louis, MO), and 2 μl RNAsin RNase inhibitor (Promega Corporation, Madison, WI, United States). The mix was then incubated for 20 min at 37°C, and subsequently for 10 min at 56°C before placing it on ice. Finally, RNA was cleaned and concentrated using the RNeasy MinElute Cleanup Kit (Qiagen, Germantown, MD) and the final product was eluted in 40 μl RNase-free water and stored at −80°C.

### DNA Sequencing

For sequencing, we shipped nucleic acid extracts on dry ice to the DOE Joint Genome Institute (JGI). Here, DNA was sheared to 300 bp using the Covaris LE220 (Covaris) and size selected using SPRI beads (Beckman Coulter). The fragments were treated with end-repair, A-tailing, and ligation of Illumina compatible adapters (IDT, Inc.) using the KAPA-Illumina library creation kit (KAPA biosystems) and 5–20 cycles of PCR were used to enrich for the final library. Sequencing of metagenomic libraries was performed on an Illumina NovaSeq sequencer to produce 2 × 151 base pair (bp) paired-end reads. Contaminant removal (human, mouse, cat, dog, microbial, synthetic, non-synthetic), trimming of adapters, and right-quality trimming of reads where quality dropped to 0 was done with BBDuk. BBDuk was also used to remove reads that contained four or more “N” bases, had an average quality score across the read of less than 3, or had a minimum length ≤51 bp or 33% of the full read length. Quality control removed between 0.2 and 9% of raw sequences, resulting in a total of 247–661 million quality-controlled reads per sample.

Trimmed, screened, and paired-end Illumina reads were read-corrected using bfc (version r181) with -k 21. Reads with no mate pair were removed. The remaining reads were assembled using SPAdes using the settings “–only-assembler -k 33,55,77,99,127 –meta.” The entire filtered read set was mapped to the final assembly and coverage information generated using BBMap with default parameters except ambiguous = random. See Supplementary Table 2 for details on software versioning and read numbers.

### RNA Sequencing

For RNA sequencing, ribosomal RNA was removed from total RNA using a Ribo-Zero^TM^ rRNA Removal Kit (Epicenter). Stranded cDNA libraries were generated using the Illumina Truseq Stranded RNA LT kit. The rRNA depleted RNA was fragmented and reversed transcribed using random hexamers and SSII (Invitrogen) followed by second strand synthesis. The fragmented cDNA was treated with end-pair, A-tailing, adapter ligation, and 10 cycles of PCR. Low input libraries were sequenced on an Illumina NovaSeq sequencer, generating 2 × 151 bp long paired-end reads. Contaminant removal and quality control was conducted in the same way as for the metagenomic reads (see above). The final filtered fastq contained 48,612,144 and 23,642,934 reads in the cryopeg and the sea-ice metatranscriptome, respectively (Supplementary Table 2).

The quality-filtered reads were assembled with MEGAHIT v1.1.2 (kmers: 23,43,63,83,103,123). For coverage information, BBMap was used to map the quality-filtered reads against the metatranscriptome assemblies, as well as against the corresponding metagenomic reference assemblies with default parameters except for ambiguous = random.

### Functional Annotation

Functional and structural annotation of the generated metagenomic and metatranscriptomic assemblies was performed through the DOE-JGI Metagenome Annotation Pipeline (MAP) (Huntemann et al., 2016). Information on pipeline, software used, and versioning is provided in Supplementary Table 2. Briefly, the pipeline first identifies structural RNAs and regulatory motifs, protein-coding genes, tRNAs and CRISPR arrays, which is followed by functional annotation of protein-coding genes by assignment to 3D fold and functional protein families using various databases. Here we focused on the assignment of KEGG Orthology (KO) Terms. The coverage information obtained from read mapping is used to calculate “estimated gene copies,” whereby the number of genes is multiplied by the average coverage of the contigs, on which these genes were predicted. The JGI-IMG Phylogenetic Distribution of Best Hits tool (Chen et al., 2019) assigns taxonomy to genes in an assembly through a homology search using LAST (Frith et al., 2010) against IMG reference isolates (high quality public genomes). Scaffold lineage affiliation is then assigned as the last common ancestor of all best gene hits on the scaffold, provided that at least 30% of the genes have hits.

### Taxonomic Composition of Metagenomes and Metatranscriptomes

We used the phyloFlash pipeline (Gruber-Vodicka et al., 2019) to screen the quality-controlled metagenomic reads for small subunit ribosomal RNA (SSU rRNA) reads by mapping against the SILVA SSU Ref NR 99 database 132 (Quast et al., 2013). The top reference hits per read were used to report an approximate taxonomic affiliation, and the mapped read counts were then used to generate an overview of community composition across samples. We used default settings with the parameters “-readlength 150 –almosteverything.” For the rRNA depleted metatranscriptomes, we used scaffold taxonomy (see above) to report community composition.

We used SPAdes (as implemented in phyloFlash) for a targeted assembly of full-length SSU rRNA sequences from the identified SSU rRNA reads. From the resulting 460 reconstructed sequences, we selected those of dominant prokaryotic community members (genera of >1% relative abundance) for multiple sequence alignment using MAFFT Q-INS-I (Katoh et al., 2019). We used the resulting alignment to calculate a Neighbor-Joining tree of genus representatives with the Jukes-Cantor model and 1,000 bootstraps. *Candidatus* Nitrosopumilus, the most abundant archaeal community member, was included as tree outgroup despite relative abundance values <1%. The tree and the corresponding community abundance data were visualized with iTOL v4 (Letunic and Bork, 2019).

### Comparative Analysis of Prokaryotic Adaptive Genomic Strategies

For comparative analyses of the functional potential encoded in the microbial communities in cryopeg brines and sea ice, we focused on molecular functions represented in the KEGG Orthology (KO) database (Kanehisa et al., 2016). Here, genes are grouped as functional orthologs of experimentally characterized proteins, and further associated with higher-level functions in the context of molecular pathways and modules. We filtered the data for eukaryotic sequences, because eukaryotes, predominantly diatoms, can be a major component of sea-ice communities in lower sea-ice sections and therefore contribute to metagenomic sea-ice data. Although eukaryotes were negligible in cryopeg brines (Supplementary Figures 2A, 3), cryopeg data were similarly filtered. We used EukRep, a classifier that utilizes k-mer composition of assembled sequences to differentiate between prokaryotic and eukaryotic genome fragments, with a minimum sequence length cutoff of 1 kb for the identification of eukaryotic contigs in our assemblies (West et al., 2018). We then subtracted the estimated KO abundances for KOs predicted on eukaryotic contigs from the total gene abundances for each sample.

To allow a direct and biologically meaningful comparison between samples, we normalized all metagenomic datasets using Metagenomic Universal Single-Copy Correction (MUSiCC) with the settings “–normalize –correct learn_model” (Manor and Borenstein, 2015). Rather than comparing the relative abundance of KOs between samples, MUSiCC estimates the average genomic copy number of a KO in a given sample using a large set of universal single-copy genes for gene abundance calibration.

As individual KOs can be associated with multiple higher-level functions, we used Evidence-based Metagenomic Pathway Assignment using geNe Abundance Data (EMPANADA) to infer the prevalence of different KO pathways and modules in each sample (Manor and Borenstein, 2017). Here, the average abundance of only non-shared KOs is used to generate support values for each pathway, and to subsequently partition the contribution of shared KOs between pathways. We used the MUSiCC-normalized KO data as input with the settings “–mapping_method by_support -use_only_non_overlapping_genes -v -remove_ko_with_no_abundance_measurement.” Abundance values for pathways and modules therefore represent the average number of genes belonging to a pathway per genome.

### Statistical Analyses and Visualization of Results

We performed all statistical analysis and visualization of results in R v3.6.1 (R Core Team, 2017). We calculated alphadiversity indices per sample using the *vegan* package v.2.5.6 (Oksanen et al., 2019) with the *diversity* function, and performed an analysis of variance (ANOVA) followed by a *post-hoc* Tukey pairwise significance test to assess the significance (*p* ≤ 0.05) of variance in observed richness and Shannon diversity between both brine environments. We performed a Principal Component Analysis (PCA) for the analysis of beta diversity patterns in our data. We used the *ampvis2* package v.2.5.8 (Andersen et al., 2018), which uses the *vegan* package v.2.5.6 (Oksanen et al., 2019) for ordination and the *ggplot2* package v3.3.2 (Wickham, 2016) for plotting. The taxonomy data has been transformed by applying a Hellinger transformation prior to ordination analysis. By the creation of a biplot and adding species scores to the PCA ordination, we inferred information about taxa and KO functions that contributed most to observed sample spread. Further, we used the Bray-Curtis distance measure on the basis of relative abundances of genus-level taxonomy and normalized KO abundances, and performed an Analysis of Similarity (ANOSIM) with 999 permutations to test whether between-group differences were larger than within-group. For the community composition heatmaps, we used the heatmap function within *ampvis2* with default parameters.

We used the *stats4bioinfo* package v1.1.0 (van Helden, 2016) to perform per-row Welch’s *t*-tests to identify genes, modules or pathways where the sample mean differed in abundance between cryopeg brine and sea-ice samples. By applying multiple testing correction, we identified significantly different features as those genes, modules or pathways that passed the false discovery rate (FDR) correction with a corrected *P* < 0.05 (Storey and Tibshirani, 2003). We visualized pathway-level results in a heatmap using the *pheatmap* R package v1.0.12 (Kolde, 2019) and used the euclidean distance measure with ward.D clustering for dendrogram generation. Results were scaled by row to display z-scores depicting the deviation from the mean. For module-level results, we generated box plots that show median and interquartile ranges of average number of genes per module per genome using the *ggplot2* package v3.3.2 (Wickham, 2016).

## Results and Discussion

### Distinct Taxonomic and Functional Diversity Patterns

**FIGURE 1:**
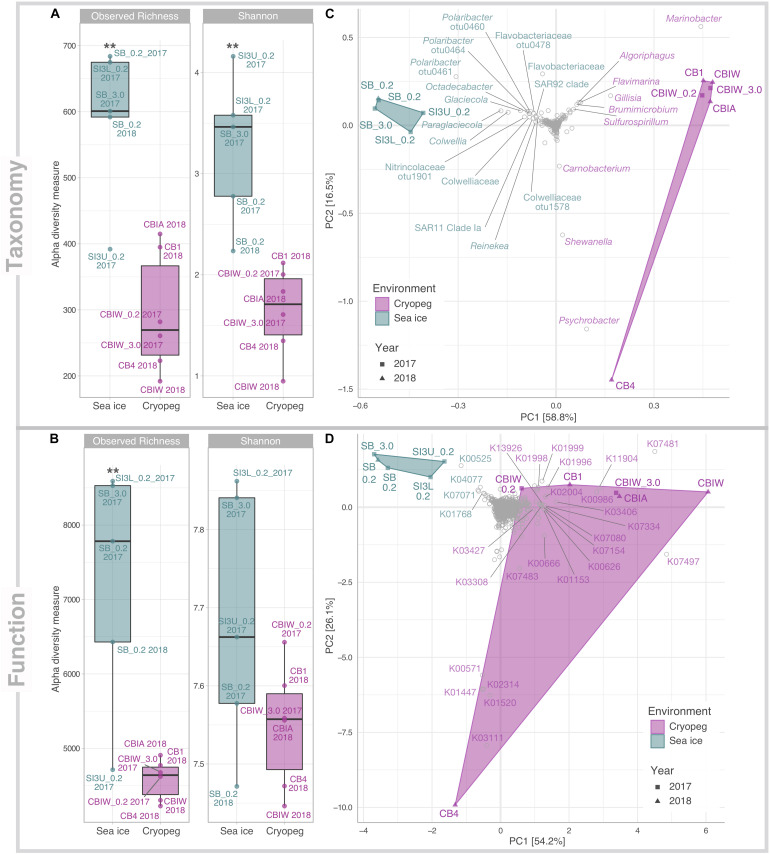
Alpha and beta diversity of prokaryotic community composition and functional potential in sea ice and cryopeg brines. Indices of **(A)** taxonomic observed richness and Shannon diversity on the basis of taxonomic assignments inferred for extracted 16S rRNA gene reads; and **(B)** observed richness and diversity of functional potential on the basis of normalized KO abundances. Asterisks indicate significant variance between means of both environments (** indicates *p* < 0.01). Beta diversity of **(C)** taxonomic composition and **(D)** functional potential was visualized through principal component analysis (PCA). The relative contribution (eigenvalue) of each axis to the total inertia in the data is indicated in percent at the axis titles. For **(C)** data were transformed initially by applying a Hellinger transformation; for **(D)** data were normalized through MUSiCC (see section “Materials and Methods”). An additional layer of information was added in the form of species scores to specify **(C)** prokaryotic taxa and **(D)** molecular functions that contributed most strongly to the variance between samples. K00525: ribonucleoside-diphosphate reductase alpha chain; K00571: site-specific DNA-methyltransferase (adenine-specific); K00626: acetyl-CoA C-acetyltransferase; K00666: fatty-acyl-CoA synthase; K00986: RNA-directed DNA polymerase; K01153: type I restriction enzyme, R subunit; K01447: N-acetylmuramoyl-L-alanine amidase; K01520: dUTP pyrophosphatase; K01768: adenylate cyclase; K01996: branched-chain amino acid transport system ATP-binding protein; K01998: branched-chain amino acid transport system permease protein; K01999:branched-chain amino acid transport system substrate-binding protein; K02004: putative ABC transport system permease protein; K02314: replicative DNA helicase; K03111: single-strand DNA-binding protein; K03308 neurotransmitter:Na + symporter, NSS family; K03406 mcp; methyl-accepting chemotaxis protein; K03427: type I restriction enzyme M protein; K04077: chaperonin GroEL; K07017: uncharacterized protein; K07080: uncharacterized protein; K07154: serine/threonine-protein kinase HipA; K07334: toxin HigB-1; K07481: transposase, IS5 family; K07483: transposase; K07497: putative transposase; K11904: type VI secretion system secreted protein VgrG; K13926: ribosome-dependent ATPase.

From our combined sampling efforts in 2017 and 2018, we obtained a total of 11 metagenomes derived from cryopeg brine samples (*n* = 6) and sea-ice brines and section melts (*n* = 5). Assemblies of individual datasets resulted in overall larger and more fragmented assemblies from sea ice than from cryopeg, with sea-ice metagenomes having, on average, more but shorter contigs, and being enriched for singleton contigs and unassembled reads (Supplementary Table 2). This difference is likely due to input from a taxonomically richer (*p* < 0.01) and more diverse (*p* < 0.01) community in sea ice than in cryopeg brines (Figure 1A). Functional alpha diversity in both environments followed similar trends, but was only significantly higher in sea ice for functional richness (*p* < 0.01) though not functional diversity (Figure 1B). Presumably the long residence time and environmental stability of cryopeg brines selected for fewer, but more functionally diverse organisms.

**FIGURE 2:**
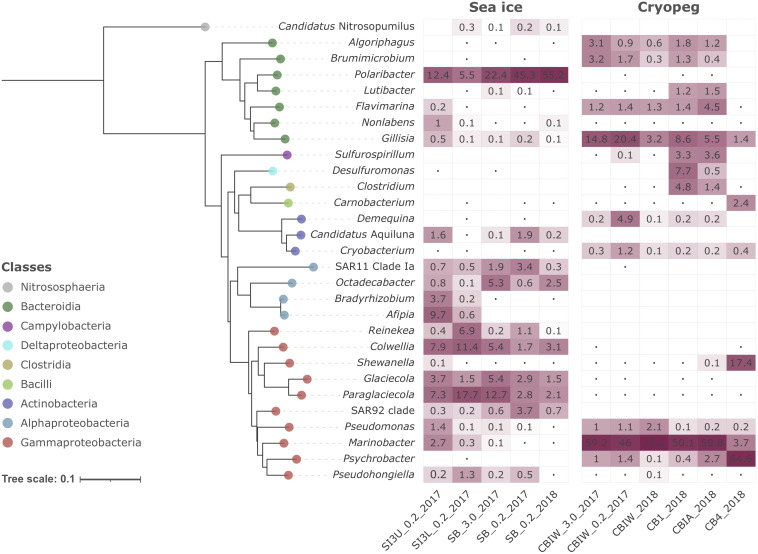
Heatmap of prokaryotic community composition in sea-ice and cryopeg brine metagenomes. Dominant genera (>1% relative abundance in at least one sample) are organized in the form of a Neighbor-Joining phylogenetic tree reconstructed on the basis of full-length 16S rRNA genes. *Candidatus* Nitrosopumilus was chosen as outgroup. The Jukes-Cantor model and 1,000 bootstraps were used for tree calculation. Dots symbolize values <0.05%; empty cells indicate no detection of that genus in a particular sample.

Compositionally, brine communities were clearly separated from sea-ice communities both taxonomically (Figure 1C, ANOSIM R = 0.93, *p* < 0.01) and functionally (Figure 1D, ANOSIM, R = 0.94, *p* < 0.01). In sea ice, the strongest drivers of the taxonomic variance from cryopeg brines were members of *Bacteroidetes*, mainly *Polaribacter* and unclassified *Flavobacteriaceae*, as well as the gammaproteobacterial genera *Paraglaciecola*, *Glaciecola* and *Colwellia*, and the alphaproteobacterial genus *Octadecabacter* and SAR11 clade (Figure 1C)—all were dominant in sea ice and near- (<0.05%) or completely absent from the cryopeg brines (Figure 2). In cryopeg brines, *Marinobacter* contributed the most strongly to the variance from sea ice (Figure 1C), in keeping with its predominance in the cryopeg samples (46–79% of the prokaryotic community) except for CB4 (4%; Figure 2). *Marinobacter* representatives have been observed and isolated from a variety of other cold and saline environments, including Blood Falls, the saline subglacial outflow from Taylor Glacier, Antarctica (Mikucki and Priscu, 2007; Chua et al., 2018; Campen et al., 2019), a cold saline spring in the Canadian High Arctic (Niederberger et al., 2010; Trivedi et al., 2020), and Arctic sea ice (Zhang et al., 2008). Experimental work on isolated members (reviewed by Handley and Lloyd, 2013), as well as genomic insights into the functional capabilities of *Marinobacter* members (Singer et al., 2011), attribute to this genus a remarkable versatility in growth and energy acquisition strategies and a high genomic potential to adapt to challenging environmental conditions, which may have enabled *Marinobacter* to compete effectively for resources and reach high relative abundance (Figure 2) despite the extreme conditions in cryopeg brine.

Cryopeg brine CB4 differed markedly from the other cryopeg brines, with *Psychrobacter*, *Shewanella*, and *Carnobacterium* as drivers of this compositional distinction (Figures 1C, 2). From previous work, we learned that CB4 was also unique in its high fraction of dividing cells (29% compared to 1.1–2.5% in the other cryopeg brines; Table 1), suggesting that the CB4 community was growing more robustly. We were able to obtain a metatranscriptome from CB4 (the only metatranscriptome obtained from cryopeg brines; Supplementary Table 1), which revealed that *Shewanella*, *Psychrobacter*, and also *Marinobacter* (despite comparably low relative abundance in the corresponding CB4 metagenome) were the most transcriptionally active members in CB4 (Supplementary Figure 2B), and together accounted for >90% of the active community.

In the sea-ice metatranscriptome (SB_2018_MetaT; Supplementary Table 1), we found an overall larger number of active taxa, but the most transcriptionally active members, *Polaribacter*, *Paraglaciecola*, *Colwellia*, and *Glaciecola* (Supplementary Figure 2B), were also among the most abundant in the sea-ice metagenomes (Figure 2). The high abundance of the animal-associated *Fusobacterium* genus to the metatranscriptome (SB_2018_MetaT) and not to its corresponding metagenome (SB_0.2_2018; Figure 2) may be a result of different treatments, as the sea-ice metatranscriptome was not prefiltered (for rapid treatment in the field), like its paired metagenome, and was thus heavily influenced by eukaryotic signals (Supplementary Figures 2A, 3). Archaea were rare in both brine environments (max. 0.2% in SB_0.2_2017, max. 0.02% in CB4_2018; Supplementary Figure 2A), which we attribute to the competitive advantage of the heterotrophic bacterial groups, known to be efficient utilizers and recyclers of organic matter, present in these brines of relatively high organic content at the time of sampling (Table 1).

Differences in oxygen levels in the two studied brine environments would also contribute to shaping the resident microbial communities and likely add to the observed strong compositional divergence. Sea ice is typically well-oxygenated, and some evidence from previous work suggests that cryopeg habitats are anoxic or microaerophilic (Gilichinsky et al., 2003; Shcherbakova et al., 2009; Colangelo-Lillis et al., 2016). We lack *in situ* oxygen measurements to verify oxygen levels at our sampling sites, but our data on community structure are consistent with these previous findings as most of the dominant cryopeg community members detected here are known to be facultative anaerobes or anaerobes, e.g., *Marinobacter*, *Shewanella*, *Psychrobacter*, *Brumimicrobium*, *Algoriphagus*, *Sulfurospirillum*, *Desulfuromonas*, *Carnobacterium*, and *Demequina*, while the dominant sea-ice members are known to require oxygen, e.g., *Polaribacter*, *Glaciecola*, *Paraglaciecola* and *Colwellia*.

Functionally, there were also clear drivers of structure across these datasets. For sea-ice communities, these included genes involved in DNA synthesis (K00525: ribonucleoside-diphosphate reductase), chaperone-mediated and stress-associated protein folding (K04077: chaperonin GroEL) (Rodrigues et al., 2008), and purine metabolism (K01768: adenylate cyclase) (Figure 1D). For cryopeg brines, strong separation resulted from transposases (e.g., K07481 and K07497) and genes that may function in modulating competition (e.g., K11904: type VI secretion system secreted protein VgrG) and defense (e.g., K03427: type I restriction enzyme M protein). Separation of CB4 from the other cryopeg brines was driven by multiple functions involved in DNA replication, synthesis and repair (K02314: replicative DNA helicase; K03111: single-strand DNA-binding protein; K01520: dUTP pyrophosphatase; K00571: site-specific DNA-methyltransferase, adenine-specific; Figure 1D), in further support of a proliferating community in CB4. The following sections explore the broader implications of these functional differences.

### Common Adaptations, Different Implementation Strategies

**FIGURE 3:**
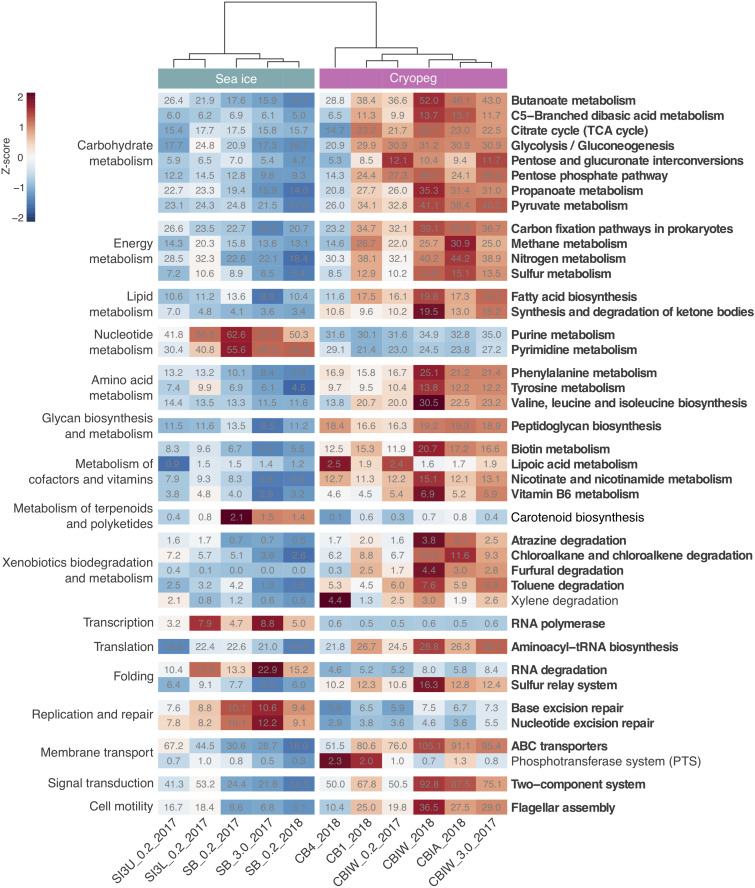
Heatmap of prokaryotic functional potential in sea ice and cryopeg brines summarized at the level of pathways involved in metabolism, genetic and environmental information processing, and cellular processes. Sample order was determined by the calculation of a dendrogram, using the euclidean distance method and ward.D clustering. Cell values display the normalized average number of pathway genes per genome in the sample (see section “Materials and Methods” for details), while cell color represents the value’s standard score (z-score), and therefore its deviation from the row mean. Displayed are pathways that were common to sea-ice and/or cryopeg microbes, i.e., detected across all sea-ice and/or all cryopeg samples with a mean abundance of >1. Those that differed significantly between brine types are in bold; pathways that did not differ significantly but showed a log2-fold difference in mean abundance of > 1 between the environments are also shown.

**FIGURE 4:**
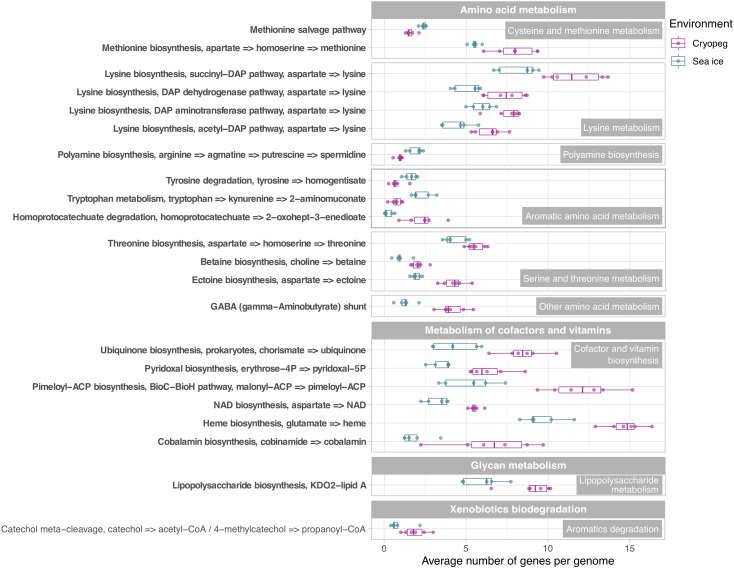
Box and whisker plots summarizing the functional potential of prokaryotic communities in sea ice and cryopeg brines for the metabolism of amino acids, cofactors and vitamins, lipopolysaccharides, as well as the degradation of aromatics. Boxes indicate the median of each group, as well as the first and third quartiles of sample distribution (the 25th and 75th percentiles). Whiskers extend from the hinge to the largest value no further than 1.5 × IQR from the hinge (where IQR is the interquartile range, or distance between the first and third quartiles). Displayed are modules that were common to sea-ice and/or cryopeg microbes, i.e., detected across all sea-ice and/or all cryopeg samples with a mean abundance of >1. Those that differed significantly between brine types are in bold; modules that did not differ significantly but showed a log2-fold difference in mean abundance of >1 between the environments are also shown.

**FIGURE 5:**
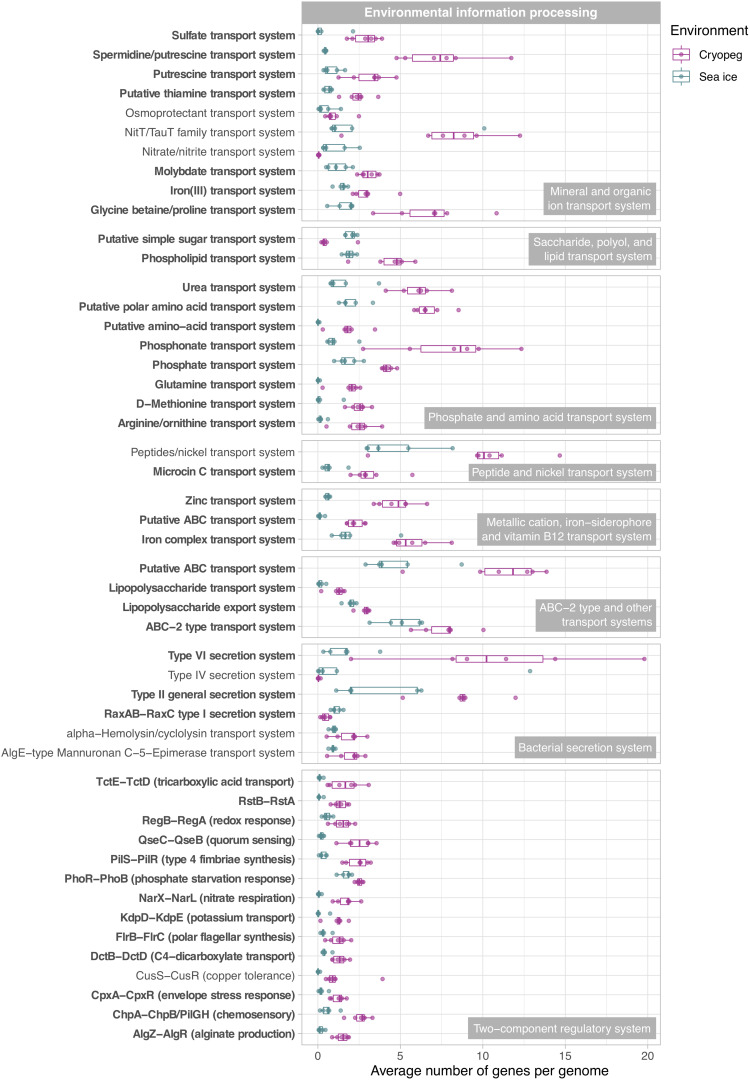
Box and whisker plots summarizing the functional potential of prokaryotic communities in sea ice and cryopeg brines for environmental information processing, including transport of compounds across cell membranes *via* ABC transport systems, use of bacterial secretion systems, and the ability to sense and respond to environmental cues *via* two-component systems. Boxes indicate the median of each group, as well as the first and third quartiles of sample distribution (the 25th and 75th percentiles). Whiskers extend from the hinge to the largest value no further than 1.5 × IQR from the hinge (where IQR is the interquartile range, or distance between the first and third quartiles). Displayed are modules that were common to sea-ice and/or cryopeg microbes, i.e., detected across all sea-ice and/or all cryopeg samples with a mean abundance of >1. Those that differed significantly between brine types are in bold; modules that did not differ significantly but showed a log2-fold difference in mean abundance of >1 between the environments are also shown.

To evaluate genomic adaptations relevant to inhabiting the two subzero environments sampled, we first identified and compared features previously reported for cold-adapted microorganisms. Our analysis approach allowed us to estimate average genomic copy numbers of genes per cell and thus to identify *in silico* features commonly enriched in both sea ice and cryopeg brines. Results confirmed that communities in both brines encode molecular functions relevant for all of the key cold-adaptation features introduced above. We also observed clear differences, however, in average gene abundance and implementation strategies. Compared to sea-ice communities, microbes in cryopeg brines invested significantly more of their genomic content in the transport of phospholipids and biosynthesis of fatty acids, peptidoglycan, lipopolysaccharides and lysine (Figures 3–5), all of which represent building blocks of the cell envelope. High numbers of cell envelope genes have been observed in other cold-adapted organisms (Médigue et al., 2005; Ting et al., 2010) and suggested to reflect a mechanism for rapid response to environmental changes and damage to the cell membrane (Tribelli and López, 2018). The permafrost bacterium *Exiguobacterium sibiricum* forms a thicker cell wall under subzero conditions as a result of higher expression of both peptidoglycan and lysine biosynthesis genes (Rodrigues et al., 2008).

A well-described mechanism to maintain membrane fluidity at low temperature involves fatty acid desaturases, which introduce unsaturated, polyunsaturated, and branched-chain fatty acids to membrane lipids (Methé et al., 2005; Ting et al., 2010; Feng et al., 2014). We found that both sea-ice and cryopeg communities encoded such enzymes at similar abundances, though the sea-ice samples displayed greater diversity of desaturases (Supplementary Table 3), likely reflective of the greater diversity of taxa (Figure 1A). In the directly collected sea-ice brines, we also observed a higher abundance of genes involved in the biosynthesis of carotenoid pigments (Figure 3). Such genes may represent another means to regulate membrane fluidity and tolerate freeze-thaw stress (Seel et al., 2020), and coincided with highest relative abundance of *Polaribacter* (Figure 2), a genus known for its yellow or orange colored members owing to carotenoid pigment synthesis (Staley and Gosink, 1999; Bowman, 2006). As carotenoid pigments are also involved in adjusting to UV light exposure and protecting against oxidative stress (Dieser et al., 2010), they may be serving multiple functions in the UV radiation-exposed sea-ice environment, but primarily cold-adaptive roles in the permanently dark cryopeg brines (Seel et al., 2020).

In both studied environments, microbes were experiencing subzero temperatures simultaneously with high salt concentrations: 112–140 ppt in cryopeg brines and 75–78 ppt in sea-ice brines (Table 1). Although the genetic potential to produce and scavenge compatible solutes for osmoprotection (Kempf and Bremer, 1998; Wood et al., 2001) characterized both systems, we observed different strategies with respect to which osmolytes were favored. While cryopeg communities were well equipped for the synthesis of betaine and ectoine (Figure 4), those in sea-ice brine showed a higher potential for synthesizing the polyamines putrescine and spermidine (Figure 4). In contrast, cryopeg brine communities appeared to depend on exogenous putrescine and spermidine, being better equipped for transport (Figure 5) than synthesis of these compounds (Figure 4), as well as for uptake of glycine betaine, proline and various other amino acids from the environment (Figure 5). Sea-ice communities encoded on average a significantly higher number of genes for putative transport systems of simple sugars (Figure 5 and Supplementary Table 4), which may serve as compatible solute or substrate (Welsh, 2000). These results from the metagenomes were largely supported by those obtained from the metatranscriptomes, with higher expression of transport systems for glycine betaine, spermidine, putrescine, and various amino acids in the cryopeg brine, and higher expression of glycerol transporters in sea-ice brine (Supplementary Tables 5, 6).

We interpret these findings to highlight the contrasting selective pressures in the two environments as follows. The continuous environmental pressure of saltier conditions in the cryopeg brines may favor diverse scavenging capabilities per cell in order to ensure sufficient acquisition of osmolytes. In contrast, microbes in sea ice may be served well enough by a narrower set of solute transport and biosynthesis capabilities, particularly if those solutes are replenished during the seasonal fluctuations that characterize the ice. The exogenous osmolytes (e.g., choline and glycine betaine) readily released by sea-ice algae during seasonal salinity stress (Ikawa et al., 1968; Torstensson et al., 2019; Dawson et al., 2020) would serve to lower the selective pressure on scavenging versatility.

### Being Economical and Planning Ahead

Microbes have evolved multiple strategies to overcome unfavorable environmental conditions, including ways to persist through periods of suboptimal nutrition, from compound storage and salvage to the use of metabolic shunts. During evaluation of our samples by epifluorescence microscopy (to obtain the counts reported by Cooper et al., 2019), we observed the presence of bright yellow circular structures evenly spaced within many of the cells in cryopeg brines (Supplementary Figure 4) though not in sea ice. We considered that these structures might be polyphosphate granules, known to serve both as versatile modulators of microbial stress responses and for energy and phosphate storage, with links to biofilm development through exopolymer production (reviewed by Seufferheld et al., 2008). We found that the key enzyme for polyphosphate biosynthesis and degradation, polyphosphate kinase 1 (PPK1) (Rao et al., 2009), was significantly more abundant in cryopeg brine than in sea-ice metagenomes (Supplementary Table 7), and only expressed in the cryopeg metatranscriptome (Supplementary Table 5). Genes for phosphate transport systems, as well as the two-component phosphate starvation response (Figure 5), were also significantly more abundant in cryopeg brines and paired with high expression. Although their precise roles require further study, polyphosphates may represent a dual strategy for both intracellular storage and stress tolerance, and the possible production of protective exopolymers (Fraley et al., 2007), which were present at much higher concentrations in the cryopeg brines than in sea ice (millimolar vs. micromolar; Table 1).

Other storage polymers of potential relevance to inhabiting subzero brines include polyhydroxyalkanoates (PHAs), nitrogen reserve polymers and cyanophycin. The relevant genes for PHA synthesis and breakdown, PHA synthase, 3−hydroxybutyrate dehydrogenase and PHA depolymerase, were present in both brine types, with no significant differences, but we observed higher gene abundances for the synthesis and degradation of cyanophycins in sea-ice communities. Cyanophycins are amino acid polymers (polyamines) that cyanobacteria and some heterotrophic bacteria, including *Octadecabacter* (Vollmers et al., 2013; Figure 2), can accumulate as cytoplasmic granules during light stress and nutrient starvation, serving as nitrogen- and possibly carbon-storage compounds (Krehenbrink et al., 2002). Biosynthesis genes for other polyamines, such as spermidine, were also more abundant in sea ice (Figure 4; see above). Genome analysis of cold-adapted *Colwellia psychrerythraea* had underscored the capacity for synthesis and degradation of polyamines (Methé et al., 2005) and its likely importance for microbial persistence in cold environments during unfavorable nutrient conditions. Other compatible solutes that contain nitrogen, including choline, glycine betaine and ectoine, can also serve as valuable organic substrates (Firth et al., 2016). Respiration of such compounds in cryopeg brines might explain the high (millimolar) concentrations of NH4^+^ measured in the brines (Table 1).

Likewise, the use of the γ-aminobutyrate (GABA) shunt may constitute an economical solution to thriving under stressful and potentially unfavorable periods of nutrient limitation in cryopeg brines, where we observed significantly higher gene abundances for this pathway than in sea ice (Figure 4). This shunt allows microbes to bypass two steps of the tricarboxylic acid (TCA) cycle by producing succinate from glutamate (Feehily et al., 2013), while also aiding in osmotic protection, as both glutamate and GABA can serve as compatible solutes (Csonka, 1989).

In sea-ice communities, we found a significantly greater potential for microbes to be economical by recycling sulfur-containing compounds through the methionine salvage pathway (Figure 4). Although ionic analyses showed that sulfur was not scarce in either brine type at the time of sampling (Supplementary Figure 5), the resident microorganisms may differ in the forms of sulfur utilized, as they differ in their potential use of compatible solutes (see above). For example, members of the SAR 11 clade, a ubiquitous marine taxon abundant in our sea-ice samples (Figure 2), lack known genes for assimilatory sulfate reduction and instead rely entirely on exogenous organic sources of reduced sulfur, such as methionine or dimethylsulphoniopropionate (DMSP), to meet their sulfur needs (Tripp et al., 2008). DMSP, often found in high concentrations in springtime sea ice (Kirst et al., 1991), provides a major source of both carbon and reduced sulfur for marine microbes (Reisch et al., 2011; Wilkins et al., 2013). The capacity for methionine salvaging would allow sea-ice microbes to exploit this additional pool of organic sulfur to fulfill their needs when DMSP is scarce. In contrast, we observed significantly higher abundances of genes for sulfate transport (Figure 5) and methionine biosynthesis (Figure 4) in cryopeg brines, and for sulfur metabolism in general (Figure 3), suggesting that economical sulfur recycling may not be required to inhabit cryopeg brines.

### Genomic Versatility and Plasticity for Competitive Advantage

While maintenance of essential cellular processes and retention of membrane fluidity for solute exchange ensure survival in subzero brines, competitive advantage may be acquired through accessory traits, e.g., the ability to sense environmental stimuli and changes, and be metabolically versatile to adjust to microniche requirements. We observed strong disparities in the accessory genomic potential encoded by communities in the two brine types, with some of the most marked differences in their environmental information-processing capacities (Figure 5). The genomes of cryopeg brine taxa encoded on average 71 genes for two-component regulatory systems, more than twice as many as sea-ice genomes (Figure 3). These simple signal transduction pathways allow bacterial cells to sense a variety of environmental stimuli and respond to changes, primarily by regulating transcription and gene expression (Wuichet et al., 2010). Specifically, our analysis showed that gene abundances in cryopeg were significantly higher for the sensing of changes in substrate availability (DctB/DctD, TctE/TctD), cellular redox state (RegB/RegA), quorum signals (QseC/QseB), envelope stress and antibiotics (CpxA/CpxR, RstB/RstA), as well as changes in chemical cues that induce the regulation of alginate, lipopolysaccharide, and hydrogen cyanide production, twitching and swarming motility, biofilm formation (AlgR/AlgZ, ChpA/ChpB), type IV pili expression (PilS/PilR), motility and biofilm formation during nitrate respiration (NarX/NarL), flagellar movement (FlrB/FlrC), potassium homeostasis (KdpD/KdpE), phosphate level regulation and virulence (PhoR/PhoB) by upregulation of bacterial secretion systems type III and VI (Figure 5).

Genes encoding two-component systems on average account for 1–2% of a bacterial genome (Salvado et al., 2015), with variation best explained by differences in genome size, lifestyle complexity and exposure to environmental fluctuations (Rodrigue et al., 2000; Alm et al., 2006). For example, the highest number of genes for two-component systems (251 genes corresponding to 3.4% of protein-coding genes) has been reported for free-living bacteria with complex life cycles and large genomes such as *Myxococcus xanthus* (Shi et al., 2008), while few have been detected in the reduced genomes of intracellular pathogens (Alm et al., 2006). By comparing gene abundances determined here (Figure 3) to the number of protein-coding genes reported for representative genomes^1^ of dominant community members (Supplementary Figure 6), we estimated the average fraction of genes for signal transduction to be 0.9% in sea-ice and 2.1% in cryopeg brine genomes. While below average abundances under the fluctuating environmental conditions in sea ice appear counterintuitive, they may be explained by the presence of several oligotrophic taxa with small and streamlined genomes, e.g., *Candidatus* Aquiluna and the SAR11 and SAR92 clades (Figure 2), for which a lack or very low numbers of two-component systems have been described (Held et al., 2019). In contrast, above average gene abundances in cryopeg microbes may represent an adaptation to diverse microbial niches, generated in part by the microscale architectures of sediment particles and exopolymers, which may develop and evolve on a shorter time frame than the larger, geophysically stable cryopeg system. A versatile suite of environmental sensing genes would enhance their capacity to exploit localized nutrient patches whenever they become available, e.g., through cell lysis, and facilitate long-term survival in this isolated environment, with little or no external input of organic matter.

Competitive advantage may also be a result of genomic plasticity mediated by mobile genetic elements and transposition processes (Bennett, 2004; Frost et al., 2005; DiCenzo and Finan, 2017). We identified transposase genes as the most abundant genes in cryopeg brines, and among the most abundant in sea ice (Supplementary Tables 7, 8), in line with previous research that identified transposases as the most prevalent and ubiquitous genes in nature (Aziz et al., 2010). Overall, the normalized abundance of KOs associated with known and putative transposases was more than twice as high in cryopeg (on average 55 genomic copies or 2.3% of all KOs) than in sea ice (23 genomic copies or 1.5% of all KOs), though differential abundances between individual types of transposases were mostly non-significant due to high within-group variation (Supplementary Table 8). Outside these systems, the number of transposase genes per genome can be highly variable, ranging from zero in the small, streamlined genomes of open ocean picocyanobacteria to over a thousand copies in large genomes of filamentous cyanobacteria (Vigil-Stenman et al., 2015), with 38 copies per genome as an estimated average when present (Aziz et al., 2010). Transposase gene copy numbers are generally higher, however, in microorganisms from extreme, stressful or fluctuating environments, e.g., deep and low oxygen waters (DeLong et al., 2006; Konstantinidis et al., 2009), permafrost and ice wedge settings (Raymond-Bouchard et al., 2018), and the dynamic estuarine waters of the Baltic Sea (Vigil-Stenman et al., 2017), as well as in dense assemblages of deep-sea biofilms (Brazelton and Baross, 2009) and particulate matter (Ganesh et al., 2014; Vigil-Stenman et al., 2017). Transposition events can be stress-induced (Casacuberta and González, 2013), causing the advantageous rearrangement, activation or enrichment of a cell’s gene content in response to environmental challenges (Aziz et al., 2010), which helps to explain their higher abundance in extreme environments. We detected active transcription of transposases in both studied brine environments at the time of sampling, but expression values in the cryopeg brine far exceeded those in sea ice, up to 46 times higher (Supplementary Tables 5, 6). In highly concentrated communities, as encountered in cryopeg brines (Table 1), frequent gene exchange and selection pressure to diversify in order to compete with neighboring cells may favor the retention of high copy numbers of transposase genes, which are at the upper end of abundances reported for metagenomic datasets (Brazelton and Baross, 2009). We further hypothesize that the presumably slow growth rates of cryopeg communities allow for transposases to accumulate, despite the possible detrimental effects of transposition to a genome through gene disruption. Gene duplication resulting from replicative transposition may also explain the observed higher gene copy numbers of several accessory features in cryopeg brine communities (Figure 5).

Previous studies have repeatedly reported a significant enrichment of transposons on plasmids, where they are often encoded together with accessory traits, but few core functions (Siguier et al., 2014; DiCenzo and Finan, 2017). We observed several genomic features in our datasets indicating the presence of plasmids in both cryopeg brine and sea ice communities, including genes for at least five of the toxin-antitoxin systems (Supplementary Table 9) that have been linked exclusively to plasmids (Jensen and Gerdes, 1995; Hayes, 2003), with several of these being actively transcribed at the time of sampling (Supplementary Table 9). Their activities can play important roles in plasmid stabilization through a process called “post-segregational killing,” where plasmid loss can result in either growth arrest or cell death and, thus, selects against plasmid-free cells (Jensen and Gerdes, 1995; Harms et al., 2018). Plasmid-carrying representatives have been reported for several of the dominant genera in both brine types^2^, including *Marinobacter* in cryopeg brines, though not *Polaribacter* in sea ice (Supplementary Figure 6). Plasmids could thus be of greater relevance in cryopeg brine than in sea ice, adding extra plasticity to the cryopeg gene pool and facilitating the close co-occurrence of diverse genotypes (and phenotypes) in this stable but likely heterogeneous environment on the microscale. Both reduced negative selection pressure on plasmids and high occurrence of transposable elements favor the acquisition of novel genes, suggesting that plasmids contribute to an organism’s ability to adapt to unique niches (DiCenzo and Finan, 2017). As gained fitness advantages can be transferred horizontally (Frost et al., 2005), the presence of plasmids in cryopeg brine communities may help to explain the higher average genomic copy numbers of individual bacterial secretion (Figure 5) and toxin-antitoxin systems (Supplementary Table 9), multidrug resistance pumps (Supplementary Table 10), and xenobiotic degradation modules (Figure 3). Future in-depth analysis of our datasets targeting plasmid sequences may provide a test of this hypothesis.

### Elaborate Competitive Strategies at High Cell Densities in Subzero Brines

Within the brine system of a frozen matrix, concentrated microbes may be competing not only for resources but also for space, and therefore could benefit from the ability to sense critical cell density, relocate, and fend off competitors. Indeed, both the sea-ice and cryopeg communities encoded genes to produce, transport and modify diverse antimicrobial compounds, mainly antibacterial (Supplementary Table 10). The use of bacteriocins appears particularly important in cryopeg brines where we observed significantly higher numbers of transporter genes for microcin C (Figure 5), encoded by the *yejABEF* operon (Piskunova et al., 2017) and fully transcribed in the cryopeg metatranscriptome (Supplementary Table 5). Bacteriocins are often produced under stress conditions such as nutrient limitation and overpopulation (Riley and Wertz, 2002), and differ from other antibiotics in their target range, as they are toxic only to bacteria closely related to the producing strain (Riley and Wertz, 2002). We found that the majority of scaffolds encoding the complete operon were affiliated with the dominant *Marinobacter* and *Psychrobacter* genera in the cryopeg brines, and could further link the comparably high numbers in sea-ice sample SI3U to the dominant *Afipia* genus (Figure 2 and Supplementary Table 11). We thus hypothesize that microcin C is a potent means for dominant community members to regulate population densities, and potentially of greater relevance in the relatively low diversity cryopeg brines (Figure 1). Besides killing target cells by inhibiting essential enzymes or damaging the inner membrane (Piskunova et al., 2017), an additional relevant role for microcin C may be as a “public good” signal, where exposure induces persistence in sensitive, non-producing cells and thereby increases community resilience to various stressors (Piskunova et al., 2017).

Similarly, the activity of toxin-antitoxin systems serves an autoregulatory purpose in response to stressors (Harms et al., 2018). We detected at least 20 different types of toxin-antitoxin systems across the sea-ice and cryopeg brine metagenomes, with the great majority present in both environments yet of higher average genomic copy number in cryopeg brines (Supplementary Table 9). Multiplicity of these systems appears to be correlated to a free-living slowly growing lifestyle, and has been previously linked to the presence of mobile genetic elements (Pandey and Gerdes, 2005), in support of our observation of higher genomic copy numbers of transposases in cryopeg brines. Among the most abundant of these systems was the HipA-HipB system, which upon antibiotic exposure exerts reversible growth inhibition that coincides with increased antibiotic tolerance, thus directly implicating this module in persister formation (Harms et al., 2018; Huang et al., 2020). The toxin HipA was significantly more abundant in cryopeg brines, with multiple copies encoded per genome, indicating a greater relevance of persistence. Others, like the MazE-MazF system, which was encoded on average once per genome in cryopeg brines (but on average only once per 12 genomes in sea ice), can ultimately induce programmed cell death in part of the population (Peeters and de Jonge, 2018). In densely populated brines, individual cell death could provide multiple benefits, from limiting the spread of viral infections to releasing nutrition for the remaining population and building blocks for biofilm formation (Peeters and de Jonge, 2018).

Strong selective pressure to evade competition in both systems is also reflected in the diversity of encoded multidrug resistance and efflux pumps (Supplementary Table 10), and potential means to modify cell envelope and cell surface. We observed a significantly higher abundance of phosphatidylglycerol lysyltransferase (*mprF*) genes in cryopeg communities, used by bacteria to alter the net charge of their cellular envelope and resist a variety of cationic antimicrobials (Ernst et al., 2009), as well as for osmoprotection (Dare et al., 2014). As phosphatidylglycerol is a major building block of *Marinobacter* membranes (Park et al., 2015; Zhong et al., 2015), including high-salt-adapted members (Zhong et al., 2015), lysylphosphatidylglycerol formation could be an important strategy of the dominant bacteria in cryopeg brines for surviving both strong competition and hypersaline conditions. In sea ice, bacteria may acquire similar protection by producing capsular polysaccharides, as genes involved in transporting and exporting capsular polysaccharide components were more abundant there (Supplementary Table 4). These EPS remain intimately associated with the cell surface, where they can act as an antibiotic (and possible viral) shield, while also aiding in surface attachment and biofilm formation (Reckseidler-Zenteno, 2012) or cryoprotection (Carillo et al., 2015).

Cell concentrations in cryopeg brines were orders of magnitude higher than in sea ice (Table 1), allowing higher frequency of direct contact between neighboring cells and likely facilitating contact-dependent competitive strategies that circumvent resistance typically provided by cell envelope modifications (Russell et al., 2014). We found significantly higher gene abundances for the type VI secretion system in cryopeg brines than in sea ice (on average six times more; Figure 5), which produces a structure often described as a “molecular speargun” that uses a contraction mechanism to fire a toxin-carrying needle into neighboring cells, inducing growth arrest or cell lysis (Russell et al., 2014). Scaffold taxonomy revealed that the vast majority of cryopeg scaffolds encoding a complete type VI system belonged to the *Marinobacter* genus, and that other members of the cryopeg communities, *Shewanella* and *Pseudomonas*, also had the capacity to use this system for attack. In CB4, where no scaffold was assigned to the dominant genus *Psychrobacter* (Figure 2), the abundance of type VI genes was much lower than in the rest of the cryopeg samples (Figure 5). In sea ice, individual components of the type VI system were found scattered on multiple scaffolds, but we could only detect the complete operon on a few gammaproteobacterial scaffolds, e.g., belonging to *Psychromonas*. Previous research has indicated that sibling cells can transfer and reuse components of the type VI secretion system (Vettiger and Basler, 2016). If also true for *Marinobacter*, this mechanism could have provided members of this genus with increased competitive strength, as both its dominance and high cell densities in cryopeg brine would have increased the number of type VI components among the population. Further, type VI secretion systems have been shown to foster horizontal gene transfer in naturally competent bacteria by releasing DNA from lysed target cells (Borgeaud et al., 2015), including genes encoding new matching sets of toxins and immunity proteins (Thomas et al., 2017). Such a mechanism could have allowed *Marinobacter* and other type VI-encoding cells to successively optimize their strategies for competitive advantage in these extreme subzero brines.

## Conclusion

**FIGURE 6:**
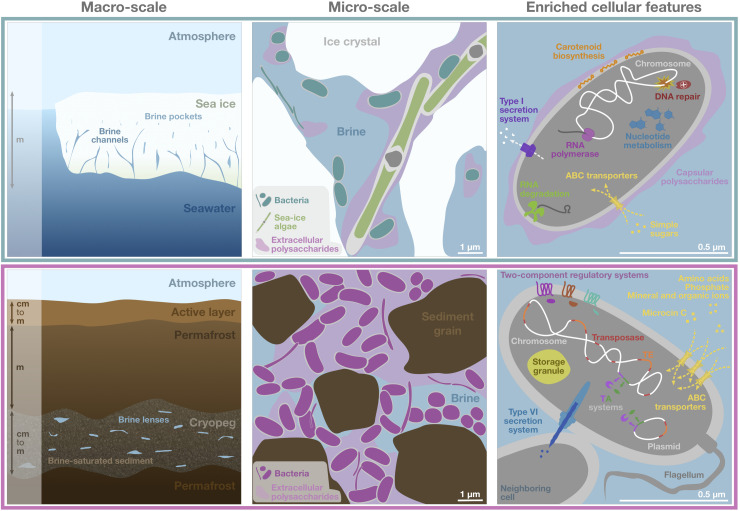
Schematic of genomic adaptations to life in subzero hypersaline sea-ice brines (upper panels) and cryopeg brines (lower panels). Sea ice, exposed to atmospheric conditions and in exchange with underlying seawater (left), is characterized by vertical gradients in temperature, salinity and nutrient availability and subject to temporal disturbances of these conditions. Its internal brine-filled network of pores and channels provides habitat for prokaryotic (primarily bacterial) and eukaryotic life (middle). Bacterial genomes in sea ice, represented by a single generic cell (right), were enriched in functions relevant for DNA replication and repair, transcription, RNA degradation, and nucleotide metabolism, as well as for membrane transport of simple sugars and the export of capsular polysaccharides, secretion of compounds through type I secretion systems and carotenoid biosynthesis. Cryopeg, a subsurface layer of unfrozen sediment within permafrost (left), is isolated from the atmosphere, permanently dark and characterized by relatively stable geophysical conditions across millennia. Brine is present in discrete lenses or as brine-saturated sediment. The cryopeg brines of this study contained high concentrations of bacterial cells, dissolved and particulate organic carbon and extracellular polysaccharides relative to sea-ice brines (middle). Bacterial genomes in cryopeg brines, represented by a generic cell and neighbor (right), were enriched in many accessory features including two-component regulatory and toxin-antitoxin (TA) systems, diverse types of ABC transporters, storage granules, and genes for flagellar assembly. An enrichment of genes for mobile elements, including transposases, transposable elements (TE), and genes exclusively present on plasmids, indicate greater genetic plasticity, while enriched type VI bacterial secretion systems and microcin C transporters attest to complex social interactions, both competitive and beneficial to the community. Canonical genes relevant for cold and salt stress were present in both communities (not shown). Scale bars are estimates for comparative purposes between panels.

Here we have presented the first metagenomic insights into the microbial ecology of cryopeg brines and the first combined exploration of functional potential and active transcription of prokaryotic communities in the subzero brines of both cryopeg and sea-ice environments. Our comparative analyses has provided novel insights into the diverse and divergent molecular strategies used by bacteria in both brine types to withstand the multitude of abiotic and biotic threats posed by their extreme environments (Figure 6) and expanded our understanding of the fundamental role that environmental stability plays in microbial adaptation to extreme conditions. A remarkable difference was the higher encoded copy number for many accessory features in cryopeg, particularly traits relevant for sensing and for transport systems. While the two brine types share the selective pressures of hypersalinity and subzero temperature, microbes within the brine inclusions face fundamentally different challenges that we believe to be central to the divergence. Compared to the contemporary sea-ice environment, the ancient cryopeg brines are far less affected by any bulk changes in their physical environment, yet diverse niches can be expected in the form of microscale chemical gradients within a dense EPS matrix and resulting from cell lysis, in many ways resembling the dynamics in bacterial biofilms (Flemming et al., 2016). Rather than a dynamic succession of individual taxa, however, the microorganisms in cryopeg brine communities appear to sustain a versatile genetic makeup that enhances phenotypic plasticity, providing them with the required tools to be highly competitive and dominant, yet also cooperative, i.e., through the sacrifice of individual members for the production of signaling compounds and the greater good nutritionally.

In the densely populated cryopeg brines, the high abundance of transposases together with high genomic copy numbers of numerous accessory traits and indications for the presence of plasmids suggest that these communities may undergo considerable genomic rearrangements, gene duplication events and horizontal gene transfer *via* mobile genetic elements. Horizontal gene transfer, also fostered by the elaborate means of microbial warfare that were detected, could support the inter- and intraspecific co-occurrence of microbial metabolisms despite overall low taxonomic diversity. The constant abiotic pressures in the cryopeg environment, the continuous competition for resources, and the limited pressure for gene deletion (assuming very slow growth) may favor the retention of genomic changes (Guieysse and Wuertz, 2012). We lack *in situ* rate measurements for the cryopeg brine communities, but our metatranscriptomic data paired with previous observations of dividing cells indicate that microorganisms were metabolically active and proliferating *in situ* despite the extreme conditions. Ongoing research aims to resolve microbial metabolisms at the level of individual genomes, and thus provide further insights into taxon-specific phenotypic plasticity, as well as high-resolution strain-level differences. This follow-on work will also address aerobic vs. anaerobic metabolisms as a means to gain insights on the role of oxygen (its presence or absence) in these extreme environments, as obtaining *in situ* oxygen measurements was not possible given the top priority to avoid introducing any contamination, particularly to the geologically isolated cryopeg brines.

In stark contrast are the challenges faced by microorganisms within the brines of sea ice. Frequent disturbance of the physico-chemical conditions imposed by hourly to seasonal fluctuations in atmospheric temperature, paired with steep vertical gradients in temperature, salinity and nutrient availability, suggests that microbial inhabitants must also be versatile to survive and thrive. However, our results imply that many members of the sea-ice community invest less in the encoding of accessory traits than those in the cryopeg communities, given the lower average copy numbers of individual features. We suggest that a distribution of capabilities across multiple community members, though potentially leading to reduced fitness of some individuals, maintains the versatility of the community as a whole, with the possible benefits of genome streamlining and more rapid replication. The community dynamics in sea ice would then follow a temporal succession, as others have documented (Collins et al., 2010; Eronen-Rasimus et al., 2016), rather than a simultaneous co-occurrence of competing taxa, with only a few members being uniquely adapted to exploit a given set of environmental conditions at a time. The comparatively higher abundance of genes relevant to DNA repair may protect replicating sea-ice taxa against DNA damage induced by exposure to radiation (Figure 6), as well as overcome periods of reduced metabolic activity.

Beyond the expected molecular adaptations that allow maintenance of vital cell functions, the survival and prosperity of bacteria in subzero brines appear to be shaped strongly by their abilities to use resources economically, to sense and respond to changes in their microenvironments, and to interact with other community members. The capability to autoregulate and induce growth arrest was a common feature and key mechanism for persistence during unfavorable conditions, including intense competition. An evaluation of the genomic potential of individual community members and efficient techniques to identify actively metabolizing members are needed to better resolve such dynamics in the future. Promising approaches include bioorthogonal non-canonical amino acid tagging (BONCAT), as preliminary results gave positive results in both brine types (Z. Zhong, unpublished data), and subsequent fluorescence-activated cell sorting (FACS) (Hatzenpichler et al., 2020) and sequencing, for specifically interrogating the fraction of brine communities active *in situ*. As environmental conditions change with amplified warming in the Arctic, particularly for historically stable cryopegs in permafrost, knowledge of current microbial dynamics in these unusual communities will be valuable to anticipating their future impacts on the larger Arctic ecosystem. One may speculate that some genes specific to surviving the current extremes and others enabling intensive social interactions, as documented here, may no longer be expressed or maintained. Communities inhabiting fresher brines in thinner sea ice will more closely resemble their source seawater communities, and the inhabitants of cryopeg brines will face new competition as permafrost thaws and the brines begin to merge with the surface environment. We can hypothesize that the strong divergence in functional potential observed between both systems described here may fade as the geophysically isolated and stable cryopeg environment transitions into a more moderate and seasonally fluctuating state. Our metagenomic analyses thus emphasize the ecological importance of habitat stability in the face of climate change.

## Data Availability Statement

All generated metagenomes and metatranscriptomes analyzed in this study were made publicly available as raw reads, quality-controlled reads, and assemblies through the IMG system (https://genome.jgi.doe.gov/portal/). For individual accession numbers per sample see Supplementary Table 2. Additional environmental metadata are available through our data archives at https://www.ocean.washington.edu/story/Deming_Data_Archives.

## Author Contributions

JD conceived the study, with input from MS and JR. JR and JD conducted the field work and sampling. JR processed the samples, performed the DNA and RNA extractions, performed all the data analyses, and wrote the first draft of the manuscript. JD and MS supported the interpretation of results and critically revised the manuscript. All authors read and approved the submitted version.

## Conflict of Interest

The authors declare that the research was conducted in the absence of any commercial or financial relationships that could be construed as a potential conflict of interest.

## Footnotes

1 Obtained from https://ftp.ncbi.nlm.nih.gov/genomes, accessed March 2020.

2 According to https://ftp.ncbi.nlm.nih.gov/genomes/GENOME_REPORTS/plasmids.txt, accessed March 2020.
