# Supplementary material for: Divergent Genomic Adaptations in the Microbiomes of Arctic Subzero Sea-Ice and Cryopeg Brines
Source: Front Microbiol. 2021 Jul 22;12:701186. doi: 10.3389/fmicb.2021.701186 (PMC8339730; doi:10.3389/fmicb.2021.701186)
Supplement: Supplementary file 1 [file Data_Sheet_1.PDF]

Supplementary Material

Divergent genomic adaptations in the microbiomes of Arctic subzero sea-ice and cryopeg brines

Josephine Z. Rapp, Matthew B. Sullivan, Jody W. Deming

Supplementary Figures

Figure S1: Principal component analysis (PCA) including sea salt control

Figure S2: Community composition at (A) domain resolution across all samples and (B) at genus resolution for metatranscriptomes

Figure S3: Detailed community composition for Bacteria, Archaea and Eukaryota

Figure S4: Microscopic images of cells in cryopeg brines with potential storage inclusions

Figure S5: Major nutrients and trace element analysis

Figure S6: Genome size and reported plasmid presence for dominant bacterial community members

Supplementary Tables

(as individual worksheets within one excel file)

Table S1: Sample overview and sampling details

Table S2: Sequence processing and output, and data accession numbers

Table S3: Detected fatty acid desaturases (FADs) and reported statistics

Table S4: Detected ABC transport systems and reported statistics

Table S5: Expression values and read counts for gene features predicted in CB4_2018_MetaT

Table S6: Expression values and read counts for gene features predicted in SB_2018_MetaT

Table S7: All detected KOs and the reported statistics

Table S8: Detected transposases and IS elements and reported statistics

Table S9: Detected defense systems and reported statistics

Table S10: Detected gene functions for antimicrobial activity and reported statistics

Table S11: Details on scaffolds that encoded a complete microcin C transport system operon

Supplementary Figures

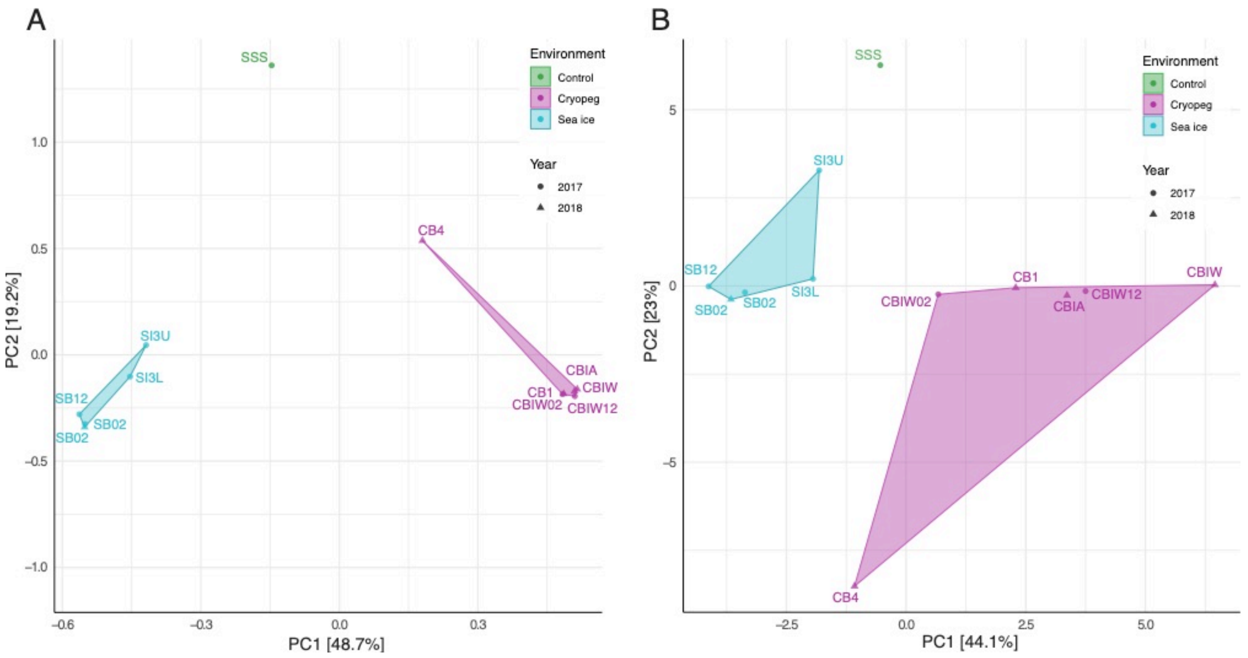

Supplementary Figure S1. Principal component analysis (PCA) of both brine environments and the sea salt solution (SSS) that was processed as a blank. PCA in (A) shows sample variation based on taxonomic composition, and (B) based on functional potential. The relative contribution (eigenvalue) of each axis to the total inertia in the data is indicated in percent at the axis titles. For (A) data have been transformed initially by applying a Hellinger transformation; for (B) KO data have been normalized through MUSiCC (see Methods).

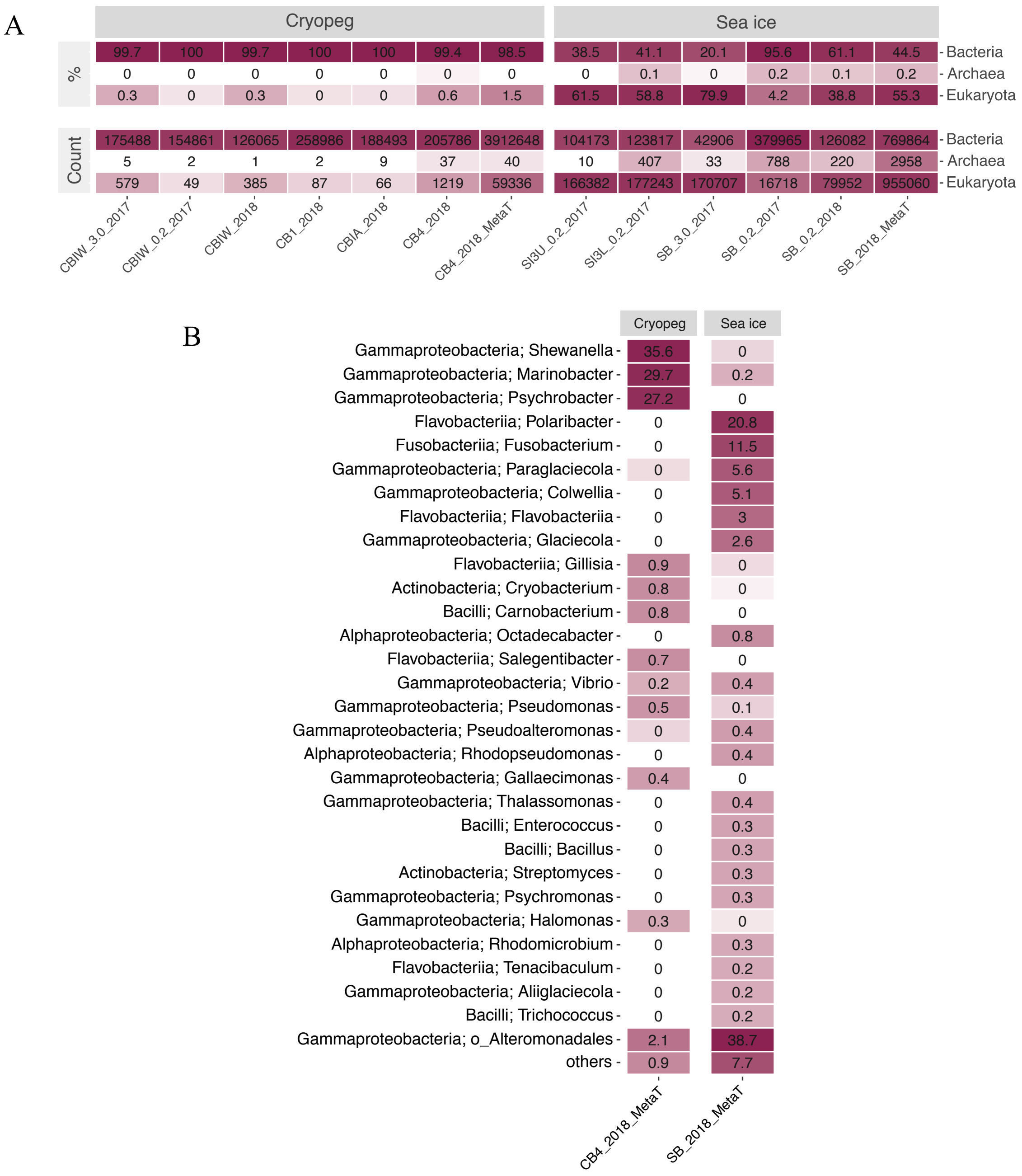

Supplementary Figure S2. Community composition in sea-ice and cryopeg metagenomes and metatranscriptomes. Composition at domain resolution is displayed both in relative abundances and corresponding read count data (A). For metatranscriptomes, community composition was inferred from scaffold taxonomy and not from extracted 16S and 18S rRNA genes, as the RNA sequencing procedures involved an rRNA depletion step. Genus-level resolution for the bacterial communities in the two metatranscriptomic datasets can be seen in (B).

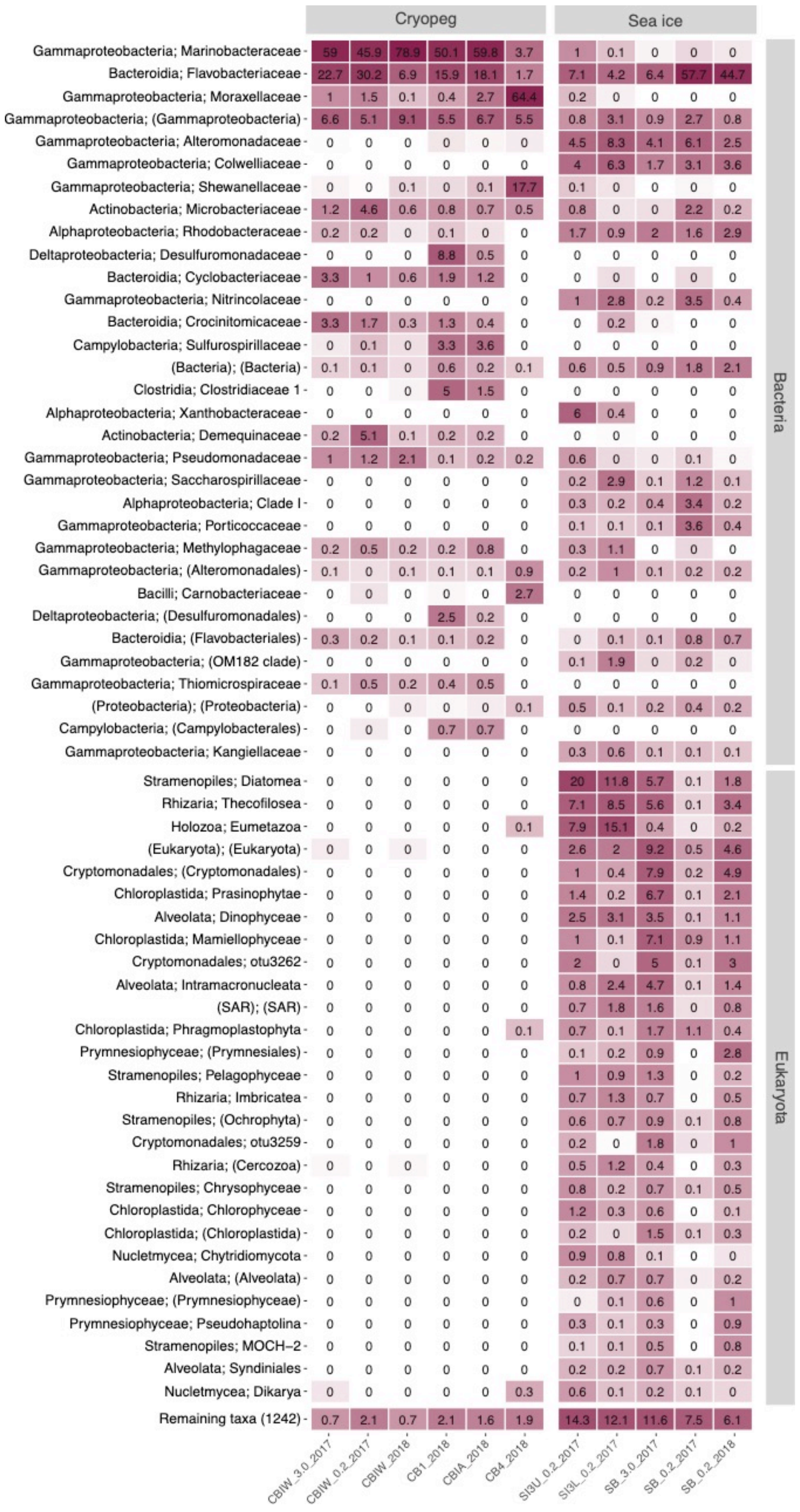

Supplementary Figure S3. Community composition in sea-ice and cryopeg metagenomes at the rank of family. Shown are relative abundance values, where the sum of all values in a column across domains is 100%.

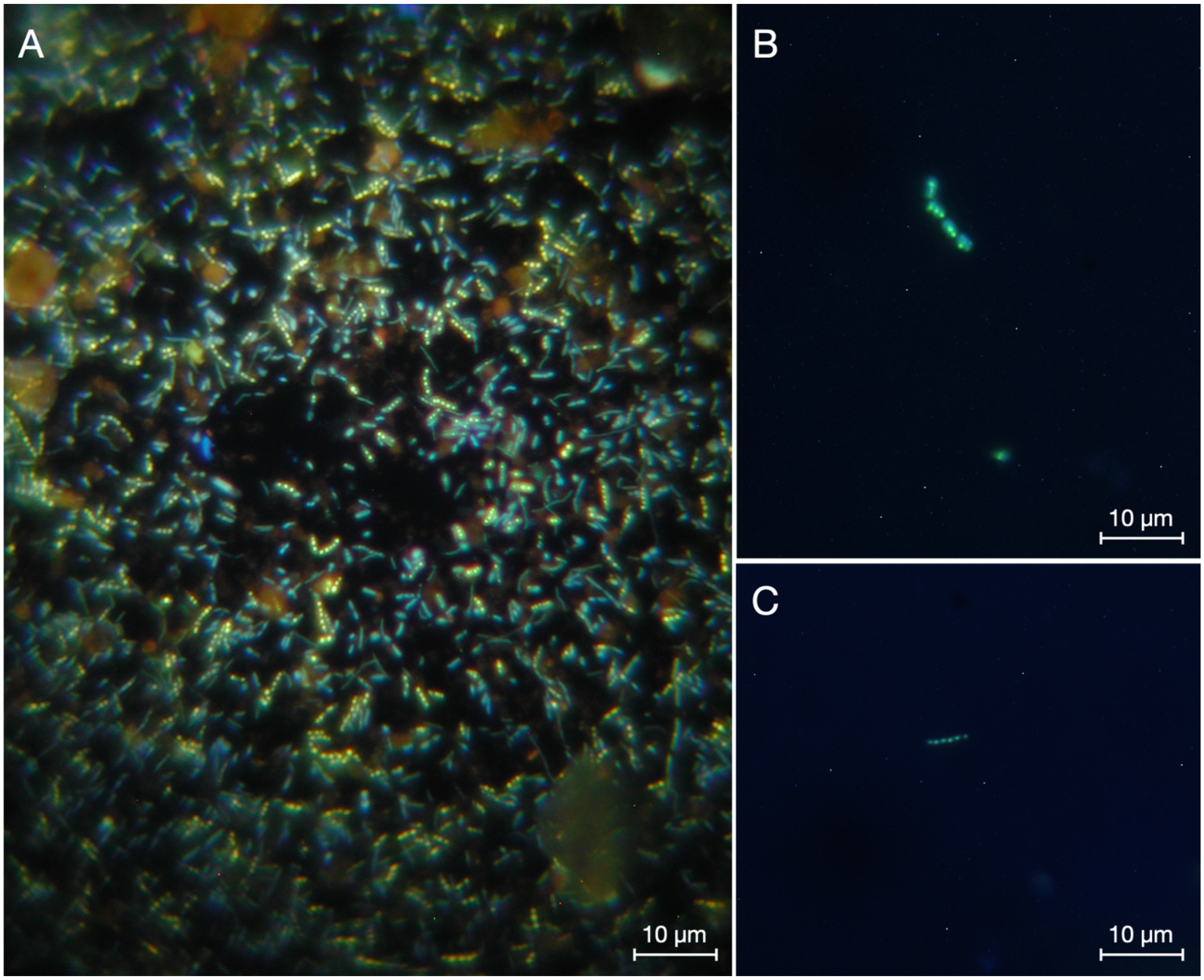

Supplementary Figure S4. Microscopic observation of potential storage granules in cryopeg bacteria. We observed circular, evenly spaced inclusions in microbes from (A) CBIW and (B & C) CB4. Samples were fixed for microscopy in 0.2-µm filtered 37% formaldehyde at a final concentration of 2%, and stored at 4° C until returned to the University of Washington. Fixed samples were then filtered onto 0.2 µm filters, cross-stained with 4′,6-diamidino-2- phenylindole (DAPI) and acridine orange, and visualized under a 1953 Zeiss Universal epi-fluorescence microscope at 1562.5× (12.5× objective × 1.25× optivar × 100× oil immersion lens), following Sherr and Sherr (1983) and Ewert and Deming (2014). Photomicrographs were obtained with a Nikon Cool-pix 990 digital camera mounted on the microscope. Sample preparation and images by S. Carpenter.

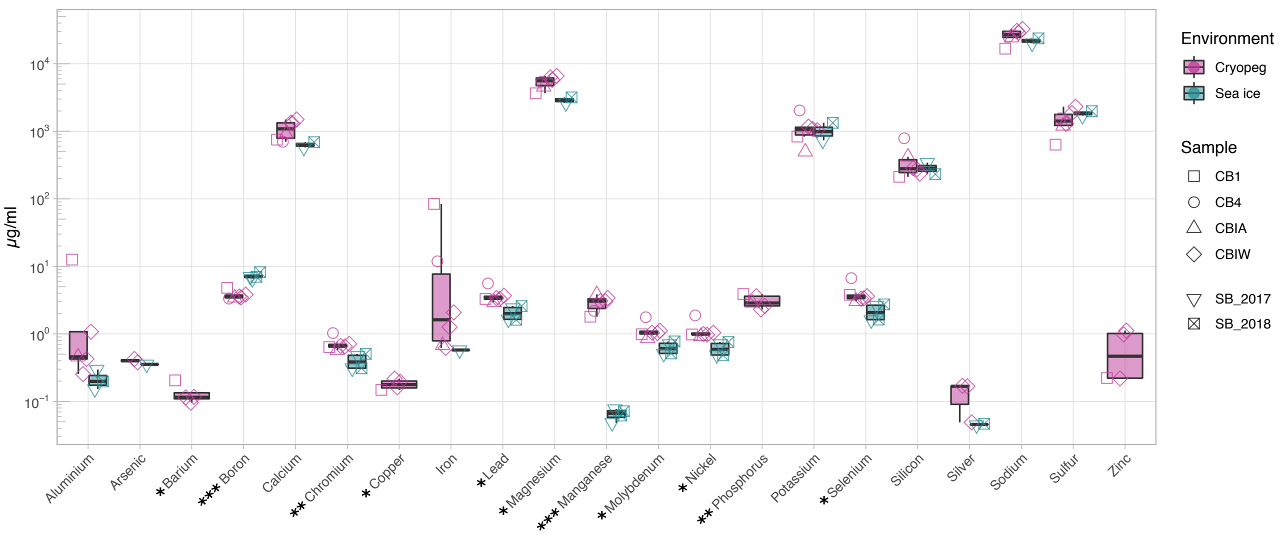

Supplementary Figure S5. Major nutrients and trace element analysis of cryopeg and sea-ice brine samples. Box plots indicate median values and interquartile ranges. Asterisks next to the metal names denote significance levels of $*p < 0.05$, $** p < 0.01$ and $*** p < 0.001$ as determined by a t-test to compare means between both environments.

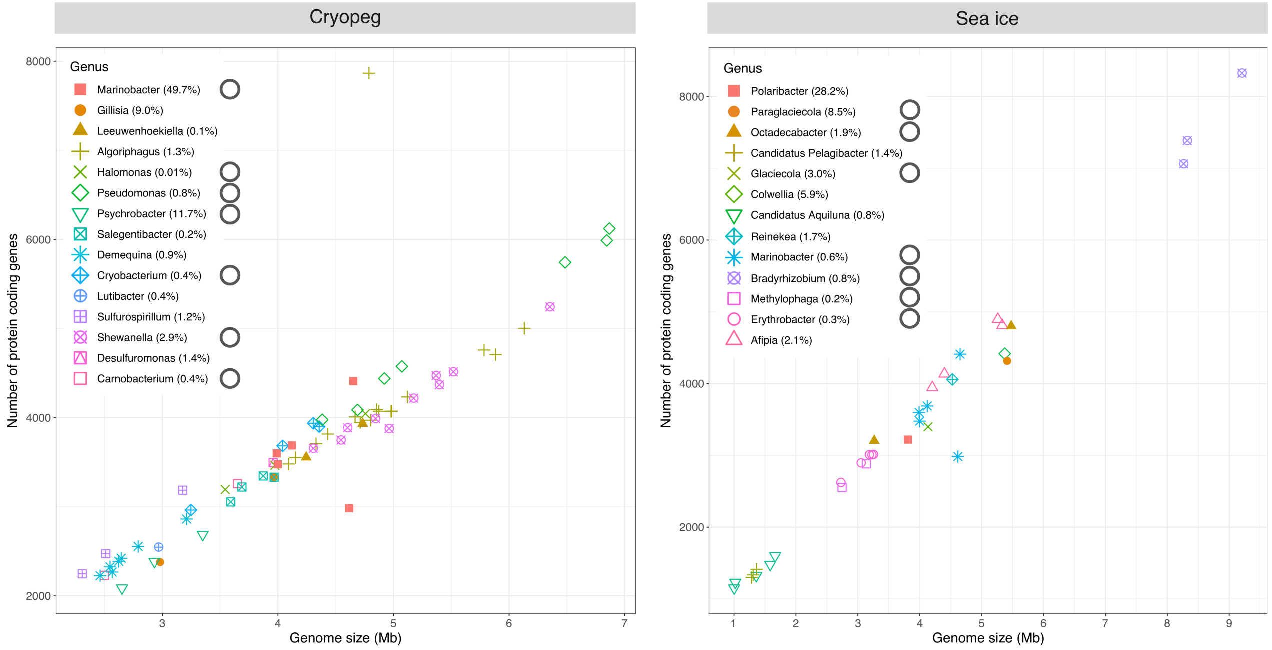

Supplementary Figure S6. A compilation of annotated proteins as a function of genome size for all dominant genera in cryopeg and sea ice. Dominant genera were those with relative abundance > 1% in at least one sample. The plot is based on data provided by NCBI genome reports (ftp://ftp.ncbi.nlm.nih.gov/genomes/GENOME_REPORTS/, accessed on February 11, 2020). If available, representative genomes (see genome reports readme file for detailed description) and whole genome sequences were used for the generation of the plot. In the absence of whole genomes, all representative genomes were used. In the absence of representative genomes, data from the total prokaryote database were screened and those with highest completion were used instead. For information on the presence of plasmids, we used NCBI's plasmid database (ftp://ftp.ncbi.nlm.nih.gov/genomes/GENOME_REPORTS/plasmid.txt, accessed in March, 2020). Genera for which representatives with plasmid sequences have been reported are marked with a black circle next to their names.

References

Ewert, M., and Deming, J. W. (2014). Bacterial responses to fluctuations and extremes in temperature and brine salinity at the surface of Arctic winter sea ice. *FEMS Microbiol. Ecol.* 89, 476–489. doi:10.1111/1574-6941.12363.

Sherr, B., and Sherr, E. (1983). Enumeration of heterotrophic microprotozoa by epifluorescence microscopy. *Estuar. Coast. Shelf Sci.* 16, 1–7. doi:10.1016/0272-7714(83)90089-6.
